# Predicting the Tolerated Sequences for Proteins and Protein Interfaces Using RosettaBackrub Flexible Backbone Design

**DOI:** 10.1371/journal.pone.0020451

**Published:** 2011-07-18

**Authors:** Colin A. Smith, Tanja Kortemme

**Affiliations:** 1 Graduate Program in Biological and Medical Informatics, University of California San Francisco, San Francisco, California, United States of America; 2 California Institute for Quantitative Biosciences, San Francisco, California, United States of America; 3 Department of Bioengineering and Therapeutic Sciences, University of California San Francisco, San Francisco, California, United States of America; University of South Florida College of Medicine, United States of America

## Abstract

Predicting the set of sequences that are tolerated by a protein or protein interface, while maintaining a desired function, is useful for characterizing protein interaction specificity and for computationally designing sequence libraries to engineer proteins with new functions. Here we provide a general method, a detailed set of protocols, and several benchmarks and analyses for estimating tolerated sequences using flexible backbone protein design implemented in the Rosetta molecular modeling software suite. The input to the method is at least one experimentally determined three-dimensional protein structure or high-quality model. The starting structure(s) are expanded or refined into a conformational ensemble using Monte Carlo simulations consisting of backrub backbone and side chain moves in Rosetta. The method then uses a combination of simulated annealing and genetic algorithm optimization methods to enrich for low-energy sequences for the individual members of the ensemble. To emphasize certain functional requirements (e.g. forming a binding interface), interactions between and within parts of the structure (e.g. domains) can be reweighted in the scoring function. Results from each backbone structure are merged together to create a single estimate for the tolerated sequence space. We provide an extensive description of the protocol and its parameters, all source code, example analysis scripts and three tests applying this method to finding sequences predicted to stabilize proteins or protein interfaces. The generality of this method makes many other applications possible, for example stabilizing interactions with small molecules, DNA, or RNA. Through the use of within-domain reweighting and/or multistate design, it may also be possible to use this method to find sequences that stabilize particular protein conformations or binding interactions over others.

## Introduction

The concept of “tolerated sequence space” – the set of sequences that a given protein can tolerate while still preserving its function at a defined level – has enabled considerable advances in understanding protein sequence-structure relationships and engineering new functions [1]. Knowing which sequences would be tolerated is important for designing for particular functions or inhibiting others [2], optimizing protein stability [3], anticipating drug resistance mutations [4], or characterizing potential evolutionary pathways [5]. Therefore, as illustrated by these examples, the ability to computationally estimate the tolerated sequence space of a protein is of both great scientific interest and practical utility. Even in cases where it is especially difficult to predict sequences optimized for a given function (for example the rate of an enzymatic reaction or the emission spectrum of a fluorescent protein), screening from a pool of predicted tolerated sequences can increase the likelihood of diversifying existing or identifying new functions [6].

To experimentally estimate the tolerated sequence space for a given protein fold, one can either use sequence alignments of orthologous proteins, or a high throughput technique such as phage display. The disadvantage of using evolutionary information is that it represents only a part of the total tolerated sequence space, and may have confounding constraints that have not yet been characterized. Moreover, simply replacing amino acids in one protein with those observed in other members of the protein's family often fails to preserve function [7], because residue interactions in proteins can be exquisitely interdependent. Phage display has been extensively used to probe the tolerated sequence space of both protein folds [8]–[10] and protein-protein interactions [10]–[16]. Phage display selects for protein binding, but through the use of a binding partner that does not interact directly with the mutated amino acids, binding can be used as a proxy for protein stability. Phage display methods are limited by the number of sequences that can be produced and analyzed. For example, allowing all 20 naturally occurring amino acid types at all positions in a standard-size protein-protein interface is generally not possible in a single screen. Therefore, computational methods that can reduce the enormous number of possible sequences to those that are more likely to be functional are extremely useful, in particular to focus libraries that can then be screened experimentally much more efficiently.

Here we provide a generalized strategy and a set of protocols for using flexible backbone protein design to predict the tolerated sequence space for a given protein fold or interaction, implemented in the Rosetta software suite for molecular modeling. Developing and, importantly, adequately testing flexible backbone protein design approaches has been a long-standing problem ( [17] and references therein). Several approaches to considering backbone flexibility in computational protein design have been described. These include sampling small random perturbations of the ψ and ϕ backbone torsion angles [18], taking backbones from a parametric family of structures [19] or using normal mode analysis [20], utilizing families of crystal structures [21] or computationally generating backbone ensembles [22]–[24], adapting dead end elimination to incorporate backbone changes [25], [26], and iterating between sequence and structure optimization [27]–[30]. Our protocol utilizes “backrub” conformational moves in Rosetta [31], [32] inspired by observations of conformational heterogeneity in high-resolution crystal structures [33]. We and others [34] have previously shown that backrub moves capture a significant fraction of the conformational variability explored by proteins to enable sequence changes [24].

We first describe the methodology and simulation protocol in-depth. Next we report key benchmarking results using phage display data. These include a new example demonstrating prediction of the tolerated sequence space of the 6 core and boundary residues in GB1, as well as the benchmarks of the generalized protocol for two systems we previously used to test variants of the computational method: the human growth hormone-human growth hormone receptor (hGH-hGHR) interface, for which approximately 1000 tolerated sequences have been determined in six phage display screens [14], and over 8000 sequences from 169 screens of naturally occurring and synthetic PDZ domain-peptide complexes [35]. The main new aspects here are the generalized protocol with a consistent set of parameters tested in several systems, detailed documentation on how to perform the computations (including all necessary source code and analysis tools as well as example input and output as part of this Rosetta collection issue), and the application of this method to the problem of predicting tolerated sequences for fold stability. We hope that providing a well-documented consistent protocol that can be applied to other systems both in a prospective or retrospective manner will stimulate further studies leading to a better understanding of transferability issues as well as scoring and sampling problems. We conclude with a discussion of current limitations as we see them and potential strategies for overcoming them, as well as future applications of the methodology described here.

## Methods

### Definitions of Sets of Amino Acid Positions

**Figure 1:**
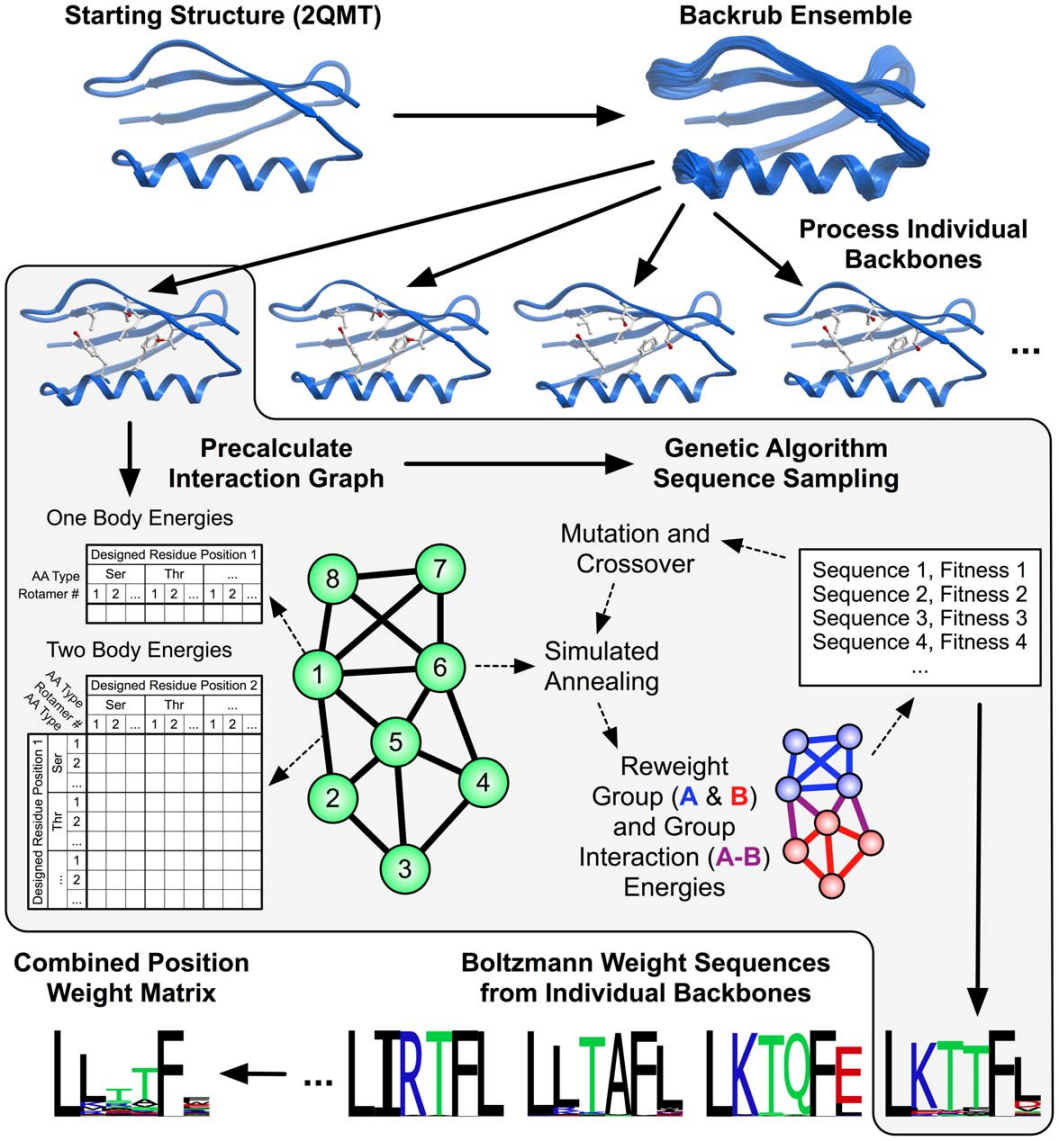
Scheme for predicting the tolerated sequences for a protein fold or interaction. The input is at least one protein structure from the protein structure databank (2QMT in the example). Rosetta first creates an ensemble of backbone conformations using the backrub method [31], then predicts sequences consistent with each conformation in the ensemble, scoring each trial sequence–structure combination using the Rosetta score12, and finally combines the sequences into a predicted sequence profile. This approach ignores potential covariation between side chains. To speed up calculations, the scoring function is split into one-body terms describing the intrinsic energy of a particular residue conformation, and two-body terms between residues; these residue-residue interaction terms are assumed to be pairwise additive. One- and two-body terms are pre-calculated and stored in an interaction graph [42] such that optimization of sequence–structure combinations for entire proteins only takes seconds using look-up tables of interaction energies. For the interaction graph, vectors of residue self-energies (one body) are stored on the vertices (green circles) and matrices of residue interaction energies (two body) are stored on the edges (thick black lines). Computed interaction energies within proteins, between proteins, or between groups of residues can be reweighted to generate custom fitness functions for specific applications. This flexibility in scoring residue groups allows modeling of separate requirements, such as those to maintain residues required in an interaction interface with a binding partner. Group and group interaction reweighting is typically only done for protein-protein interactions. (For the monomeric GB1 domain shown here, no reweighting was applied.)

The protocol and methods described here (Figure 1) aim to identify the amino acid types that can be tolerated at a given set of positions while still preserving protein fold stability and function (most commonly represented as binding). There are two general stages of the protocol: (1) creation of a set of protein backbone conformations (ensemble generation), and (2) prediction of sequences consistent with the ensemble conformations. The input to the protocol is at least one protein structure in PDB format and a definition of residue positions. There are three sets of sequence positions that can be defined: The first set of amino acids includes those that are mutated prior to ensemble generation in stage (1) and often remain the same for all subsequent simulations. These positions will be referred to as the “premutated” positions. Definition of premutated positions is optional. If no positions are chosen, the input sequence will be used for ensemble generation. The second, most important set of positions are those that can vary their amino acid type in stage (2); these have to be defined by the user and will be referred to as the “designed” positions. For each designed positions, a set of considered amino acid types can be defined, as described in the “Detailed Workflow” section below. A final set of amino acids includes those whose conformations (but not amino acid types) change during sequence scoring in step (2). This set will be referred to as the “repacked” positions and is often a superset of the “premutated” positions. These positions can be determined by the user or automatically chosen by the protocol. The predicted tolerated amino acid types at the designed positions will depend on how many other positions are allowed to vary simultaneously (for example, allowing residues in a surrounding shell to be repacked may help to accommodate different amino acid choices at designed positions). For all of the results reported here, as well as a in previous study [35], residues chosen for repack included all those with a C-alpha atom with 10 Å of the C-alpha atom of a designed position. This is the current default if repacked positions are chosen automatically by the protocol. Smaller sets of repacked positions can be used to restrict sequence diversity and simulate more conservative changes closer to the starting sequence and conformation, or to reduce the computational time required for the algorithm.

### Phage Display Datasets Used for Testing

Our study uses three datasets where a considerable number of tolerated sequences (not just a few) in a given system had been determined experimentally by phage display. The first test dataset investigated effects of sequence variations on the stability of the B1 domain of protein G (GB1) by using phage display to screen a 20 amino acid library for 6 total residues (3 core and 3 boundary) [9]. The second set, one of the largest phage display studies on protein-protein interactions, involved the human growth hormone (hGH) and human growth hormone receptor (hGHR) [14]. Through 6 separate phage display experiments randomizing 5–6 positions each, 35 amino acid positions on hGH were sampled to determine tolerated sequence space for hGHR binding. The third set is taken from a study that has determined the peptide sequence space tolerated for binding to 82 naturally occurring PDZ domains and 91 PDZ single point mutants [15].

### Input Structures

All GB1 simulations were started using PDB code 2QMT [36], which had a resolution of 1.05 Å, the highest available to date. The designed sequence positions were allowed to sample any of the 20 canonical amino acids and included residues 5, 7, 16, 18, 30, and 33. For the 56 residue GB1 domain, the repacked residues included all but 22–24, 40, 42, and 46–49 (i.e. 47 out of 56 residues). All hGH/hGHR simulations used a 2.6 Å resolution structure with PDB code 1A22 [37]. PDZ/peptide simulations used the input structures previously reported [35]. For hGH/hGHR and PDZ/peptide simulations, the designed sequence positions were allowed to sample any amino acid but cysteine.

### Backrub Ensemble Generation

During the first stage of the prediction protocol, an ensemble of backbone structures is generated using backrub Monte Carlo simulations [31], [35]. Both the backrub simulations and sequence sampling were implemented in the Rosetta 3 software suite [38]. The move set consists of 75% backrub backbone moves, 22.5% chi angle moves biased by the amino acid rotamer probabilities observed in the protein structure databank [39], and 2.5% uniformly sampled chi angle moves. Moves are accepted or rejected with the Metropolis criterion [40] using a *kT* of 0.6. After 10,000 moves are applied, the lowest energy structure from the simulation is output for the next stage of sequence sampling. For the results presented here, 200 backbones were generated from independent backrub Monte Carlo simulations for each starting structure. The exception was the hGH/hGHR predictions, which used 100 backbones to match the number of structures used previously [23]. Using fewer backbones will generally produce reasonable results, but exhibit stochastic variation. Figure S1 gives estimates of the variation as a function of the number of backbones based on a benchmark using 2000 backbones and approximately 240 million sequence scores. Predicted ranks of selected amino acid types are generally more robust than predicted amino acid frequencies. Figure S2 illustrates the dependence of prediction performance on the number of backbones. Predictions using less than 20 backbones show reduced area under ROC curve scores.) If possible, at least 100 backbones are recommended for results more robust to stochastic variation (Figure S1). For the scoring metrics summarized in Table 1, the average standard deviation over three runs when using 100–200 backbones was between 0.4–1.9% of the dynamic range of each measure.

**Table 1:** Summary of tolerated sequence prediction performance on different datasets using the generalized protocol described here.

		Residue positions	Bits of information		Fraction Top 5 (%)			
	Proteins		Phage display	Predicted		AAD (%)	AUC	Rank Top
GB1 (*kT* = 0.23)	1	6	1.58	2.66	56.9	5.61	0.74	6.17
GB1 (*kT* = 0.59)	1	6	1.58	0.89	54.2	4.05	0.71	7.17
hGH/hGHR^1^	1	16	1.19	3.58	59.3	7.46	0.75	6.00
hGH/hGHR^2^	1	35	0.89	3.24	41.9	7.48	0.64	7.72
PDZ/Peptide	5	25	3.11	2.82	81.7	4.16	0.87	2.84
PDZ/Peptide^3^	5	25	3.11	3.06	82.0	3.67	0.88	2.76

1 16 designed hGH amino acid positions as defined in [23] and shown in Figure 3.

2 All designed hGH amino acid positions shown in Figure S4.

3 Performance metrics based on position weight matrices from Smith & Kortemme 2010 [35].

Scoring metrics are used as defined previously [35]. Fraction Top 5 gives the average fraction (for every position) of amino acids with phage display frequencies ≥10% in the predicted top 5 ranked amino acids. AAD gives the average absolute difference in amino acid frequency between prediction and phage display. AUC gives the area under receiver operator characteristic curve, with true positives defined as those with phage display frequencies ≥10%. Rank top gives the average rank of the most frequently observed amino acid in phage display. The table gives results from one set of predictions as described in Methods. To gauge the variability, we repeated the predictions three times and calculated the standard deviation of the scoring metrics. The absolute standard deviations and dynamic ranges are 0.4/4.32 (Bits Predicted), 1.9/100 (Fraction Top 5), 0.4/10 (AAD), 0.006/1 (AUC), and 0.2/19 (Rank Top). As a percentage of the dynamic range of a given metric, the average standard deviations (over the first 5 rows) were: 0.9% (Bits Predicted), 1.9% (Fraction Top 5), 0.4% (AAD), 0.6% (AUC), and 1.1% (Rank Top).

The conformational variation between different polypeptide backbones modeled by the backrub method is generally small, and using larger variation often leads to flat profiles that do not agree well with experimental data [24]. For all backrub ensembles used here, the average C-alpha atom RMSD from the starting structure was 0.4–0.9 Å.

By default, the starting sequence in the input PDB is used when the entire protein structure is sampled in the fixed-sequence backrub Monte Carlo simulations in stage (1). However, there are several circumstances in which a user may want to change the sequence of the input structure prior to ensemble generation. For example, it may be desirable to mutate residues to more closely represent the experimental system. Also, experimental data may suggest that another amino acid sequence shows greater function than the sequence in the starting structure. As shown in a previous study [35], mutating the starting structure to that sequence prior to ensemble generation improves prediction performance.

Such mutations can be made manually prior to backrub Monte Carlo or done automatically as a preprocessing step of the simulation. If the automatic option is used, the side chain conformations of the mutated residues and all other residues are optimized using simulated annealing [41]. If desired, iterative minimization can be applied by including progressively more degrees of freedom in three stages (first chi angles only, then chi/phi/psi angles, finally chi/phi/psi angles and rigid body degrees of freedom).

### Designed Position Sequence Scoring

Before any sequences are scored, a graph of pairwise interaction energies between all possible conformations of all allowed amino acids is precomputed [42]. The first step of scoring a given sequence is to determine the conformations of side chains that minimize the score of the entire structure. We term this score the “raw Rosetta score”. This is done using Monte Carlo simulated annealing [41]. Once that conformation is identified, the interaction energies between and within user-defined groups of residues, often individual protein polypeptide chains, are calculated. The actual total fitness score of a given sequence is a user-defined linear combination of the self-energies and interaction energies between these groups of residues. We term this score the “reweighted Rosetta fitness score”. For the dataset of PDZ domain-peptide complexes, the optimal weights were found to be 1 for the intermolecular PDZ-peptide interaction energies, and 0.4 for the intramolecular score [35]. We used those same weights for the hGH/hGHR interaction energies. Varying these weights in a grid search showed that these parameters are transferable to the hGH system, where they produced nearly optimal fits to the phage display data (Figure S3). For the GB1 protein fold stability dataset, only the intramolecular weight was applicable, which was kept at 0.4.

The general protocol described here for all three datasets uses the default “score12” energy function in Rosetta 3, with its implementation in the 3.2 release. The only modification to the default score12 energy function was to increase the reference energy of histidine by 1.2 score units, as was done previously for PDZ/peptide specificity prediction [35]. Histidine reweighting was found to improve performance across all three datasets tested here. Other than histidine reweighting, the previous scoring function used for PDZ-peptide specificity prediction [35] differed from score12 in a number of ways: First, the Ramachandran and omega angle energy terms were turned off. (Because omega angles were never varied during the simulations, the omega energy term had no effect.) Second, the short-range backbone-backbone hydrogen bond and the amino acid probability given phi/psi terms were doubled. Third, turning off environment dependent hydrogen bonding was found to improve performance for PDZ-peptide specificity (it is on per default in standard in Rosetta 3). The first two differences to the published method [35] listed above, namely the addition of two terms and the change of two weights, are part of a “score12 patch” that is standard in Rosetta 3 methods using score12, but was not used for the PDZ-specificity prediction [35]. A discussion of the historical reasons for the bifurcation of the “standard” and “score12” weights is included in supporting information (Text S1).

### Genetic Algorithm Optimization

Sequence sampling proceeds using a genetic algorithm independently on each backbone in the ensemble. The initial population is generated by selecting random sequences from the user-defined set of allowed amino acids at the designed positions. In addition, a single population member is generated that contains the sequence from a single simulated annealing call where all possible amino acids are allowed (i.e. the sequence with the best raw Rosetta score). The population size for each generation is 2000 sequences and 5 total generations are produced, including the initial population. This results in slightly less than 10,000 sequences scored for each backbone. If 200 backbones are generated, this will result in up to 2*10^6^ sequence scores, which is within an order of magnitude of the theoretical size of the 5 and 6 amino acid libraries (3.2*10^6^ and 6.4*10^7^ sequences, respectively) used for experimental screening in the GB1, hGH/hGHR, and PDZ systems. In contrast to phage display, however, 4 out of 5 generations of sequences are not selected randomly from all possible combinations, but are increasingly enriched in later generations using an applied fitness function. Changing the number of generations to 30 was previously shown to produce equivalent results [23].

For the genetic algorithm the reweighted Rosetta fitness score is used to determine the fitness for each sequence. For every new generation of the genetic algorithm, the best fitness sequence is automatically propagated to the next generation. The remaining sequences are generated by crossover and mutation of parental sequences from the previous generation. Parental sequences are selected by tournament selection, in which two random sequences are chosen, and the sequence with the best fitness is chosen to be a parent. Half of the new population members are generated by crossover, in which two parents are chosen and the identity of each amino acid is randomly selected between the two parental sequences. Unlike physical DNA crossover, there is no linkage between sequence positions close to one another. The other half of the new population members are generated by mutation, in which a single parent is chosen and each of its amino acids is mutated with a 50% probability.

While our predictions agree reasonably well with experimental data, undersampling of sequence space and trapping in local minima are possible caveats of the applied optimization algorithms. Other sequence optimization methods could be compared to our results, such as approaches that are guaranteed to find the global minimum energy sequence [43]. Along these lines, we have found that predicted sequences using Rosetta Monte Carlo optimization are similar to results of an approach that finds all low-energy sequences within a given energy threshold of the global minimum of the Rosetta scoring function ( [44] & unpublished results). We therefore believe that inaccuracies in scoring and the inability to more accurately sample backbone variation upon sequence changes are more significant contributors to the remaining discrepancies with experimental data than fixed-backbone sequence sampling issues.

### Sequence Processing

The sequences output by the genetic algorithm are processed into a single position weight matrix (PWM) by first calculating a PWM for each individual backbone, and then merging the PWMs together. Individual backbone PWMs are calculated by Boltzmann weighting (*w = e^ΔG/(kT)^, w*: sequence weight, *ΔG*: reweighted Rosetta fitness score, *kT*: Boltzmann factor) each of the individual sequences and calculating residue frequencies. The default Boltzmann factor used here was 0.228, as determined previously [35]. The Boltzmann factor can be changed by the user (see accompanying protocol capture). PWMs are merged together with the assumption that all backbones are equivalent. The contribution of individual backbones is not weighted by their total scores because the total energy of a backbone can be largely determined by structural features distant from the designed region, which could add considerable noise. Instead, to generate a merged PWM, the median frequency for every position/amino acid type element across all backbones is calculated. Taking the median is more robust to outliers than taking the mean or weighted mean. Users can alternatively use any percentile cutoff they wish (in the accompanying protocol capture postprocessing script), with the 50^th^ percentile being equivalent to the median. While PWM analysis ignores correlations between sequence positions, a similar analysis could be done using the Boltzmann weighted sequences to calculate residue co-occurrence at two or more positions.

### Phage Display Data

**Figure 2:**
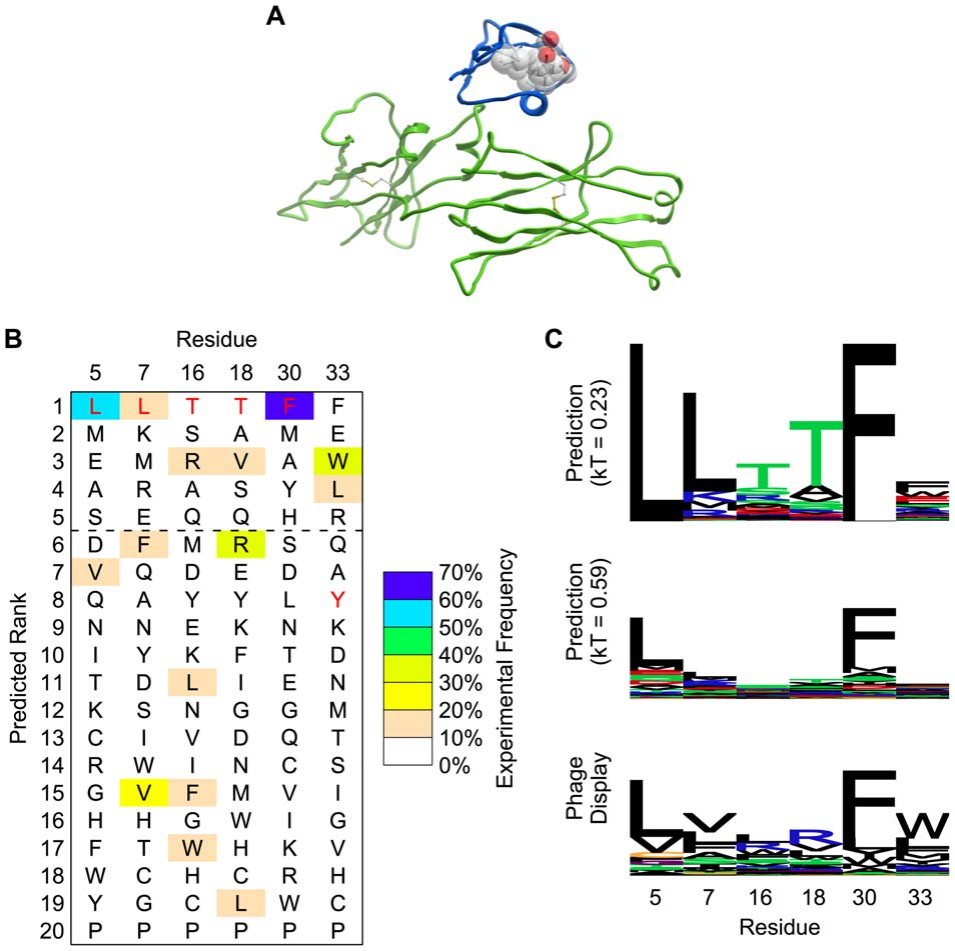
Prediction of tolerated sequences for GB1 fold stability. Frequently observed amino acids in phage display are enriched in the GB1 prediction. **A.** The structure (PDB code 1FCC) of Streptococcal GB1 (blue) is shown bound to the Fc domain of human IgG (green). The core and peripheral residues that were randomized in phage display are shown with sticks and transparent spheres. The side chain atoms (starting at C-beta) of these amino acids are at least 7 Å away from any atom of the Fc domain, making residues selected at these positions unlikely to interact directly with the Fc domain. **B.** Amino acids are ranked individually for each sequence position by computationally predicted frequency (using the Boltzmann factor *kT*  = 0.23, as described in the main text). Wild type residues, which were used in protein ensemble generation, are shown in red. The dashed line indicates a typical cutoff of picking the top 5 amino acid choices at each position. **C.** Sequence logos (LOLA, University of Toronto) are shown for predictions with two different Boltzmann factors. The relative degree of specificity (in terms of bits of information, y-axis) shows good correspondence between prediction and phage display. Increasing the Boltzmann factor lowers the overall specificity and brings the absolute frequencies closer to phage display.

Raw sequencing data (Andrea G. Cochran, personal communication) from round three of phage display of the Streptococcus GB1 domain using the human IgG Fc domain as bait [9] included 185 sequences. Sequences were excluded that contained ambiguous reads, early stop codons, and mutations at sites other than those explicitly varied, leaving 171 total sequences and 167 unique sequences. For the hGH/hGHR example, phage display frequencies were taken from Figure 2 of the authors' publication [14]. Erbin PDZ frequencies were used as previously described [35].

### Detailed Workflow

The following is a detailed description of the steps that need to be taken to apply the described method to another system, or reproduce the results of the analysis done here. The protocol capture accompanying this manuscript contains all the input files, command lines, and postprocessing scripts for replicating the computations, figures, and tables given here. (Text S1. Background on the “standard” and “score12” Rosetta energy function weights

 (Dataset S1, with any future updates available at http://kortemmelab.ucsf.edu/data/)

#### Select and prepare input structure

The input structure should be a crystal structure, NMR structure, or high quality homology model. If multiple structures are available (e.g. an NMR ensemble), the input structures should be placed into separate PDB files for input into the *backrub* application. Input of multiple structures can be facilitated by the *backrub_seqtol.py* script if they are numbered sequentially starting at 1, for instance *PDB_01.pdb*, *PDB_02.pdb*, etc.

#### Determine which amino acids will be premutated, designed and repacked and create resfiles

Each of these sets of residues is described above. If there are no premutated residues, a *backrub* resfile is unnecessary. If there are, those should be placed as *PIKAA X* (picking the desired amino acid X by one letter code) in the *backrub* resfile, with the default behavior for all other residues specified as *NATAA* (i.e. sample side chain conformations while preserving the native amino acid type).

A resfile is required for the *sequence_tolerance* application and should contain the designed and repacked sets of residues. Designed residues should use either *ALLAA* (all amino acids) or *PIKAA XYZ…* (picking the allowed amino acid residues with one letter codes X, Y, Z, etc.). Repacked residues should use *NATAA* and nonrepacked residues should use *NATRO* (native rotamer). A convenience script, *seqtol_resfile.py*, will generate a resfile for an input structure and a given set of designed residues, automatically determining the repacked residues having C-alpha atoms within 10 Å of the designed residue C-alpha atoms.

#### Determine whether to minimize after premutation and create movemap file

If premutated residues are specified using the *backrub* resfile, an optional stage of minimization is recommended and can be enabled after the premutation step but before the backrub Monte Carlo simulation. To do so, a movemap file (specified using the *-backrub:minimize_movemap* option) must be created which specifies the sidechain, backbone, and rigid body degrees of freedom to minimize. This was done, for example, in the case of the Erbin mutant V83K to minimize all side chains and the most N-terminal backbone dihedral angles of the peptide. If backbone dihedral angles or rigid body degrees of freedom are minimized, care should be taken with the fold tree; information on the fold tree is given in the Rosetta 3.2 manual and Leaver-Fay et al [38].

#### Determine whether to sample phi/psi angles directly and create movemap file

While not used for any results published here or elsewhere to date, it is possible to have the backrub Monte Carlo procedure also make small direct perturbations to phi/psi angles of the protein. To do so, a movemap file (described in the Rosetta 3.2 manual) must be provided using the *-in:file:movemap* option. In addition, the *-sm_prob* option, which gives the probability of making a “small” combined phi/psi move [45], must be given a positive value. The fold-tree warning above about minimizing backbone degrees of freedom applies to backbone perturbations as well.

#### Create backrub ensemble

The *backrub* application can be run once and produce many different backbones, each starting from the original specified structure. As an alternative, the *backrub* application can be run separately each time a new ensemble member is required. The *backrub_seqtol.py* script does this and renames the resulting structures as if they came from a single execution of the *backrub* application. On a heterogeneous cluster, this stage took 20 seconds to 10 minutes per backbone for the results published here.

#### Determine appropriate fitness function and score a large number of sequences

The *sequence_tolerance* application is used to score a random selection of sequences that are increasingly enriched in those that conform to the prescribed fitness function, whose coefficients are specified using the *-seq_tol:fitness_master_weights* option, which is fully described in the Rosetta 3.2 manual. The fitness function individually weights interactions between and within sets of residues defined by the PDB chain identifier. The sequence scoring process took 15 minutes to 5 hours per backbone for the results published here.

#### Post-process sequence scores

Post processing of the results is done using an *R* [46] script in the *sequence_tolerance.R* file. The function used, *process_specificity()*, takes several parameters. The first parameter, *fitness_coef*, allows the user to specify a vector of coefficients for the fitness function used in postprocessing. The second parameter, *temp_or_thresh*, allows the user to specify the Boltzmann factor (temp) or threshold cutoff value above the minimum fitness (thresh). The third parameter, *type*, determines how sequences are weighted and *temp_or_thresh* is interpreted. Sequences are either weighted using the Boltzmann equation ("boltzmann"), or a binary threshold cutoff ("cutoff"). The final parameter, *percentile*, gives the percentile to use for merging frequencies from multiple backbones together. The default value, 0.5, corresponds to the median frequency across all backbones.

Good results can still be obtained even if the genetic algorithm uses weights for tournament selection that are slightly different from those used for final sequence scoring. For instance, in a previous PDZ peptide specificity study [35] and the results reported here, the genetic algorithm used a ratio of 1∶2 between the weights of intramolecular and intermolecular interactions, while the final sequence scoring was done using a ratio of 1∶2.5. The user thus has the flexibility to make small perturbations to the weights during post-processing without running the whole algorithm again.

### Caveats and Factors Not Taken into Account

For the case of interface optimization, residue-residue interactions across the interface are upweighted in lieu of explicitly calculating the scores of the two partners separately and in complex. This was done in part for computational efficiency and in part because separate calculation of scores was found to add noise to interface ΔΔG prediction (unpublished results). If the designed residues change their conformations in energetically significant ways when not in complex, the algorithm will neglect those contributions to binding affinity. Also, the contribution of conformational entropy changes is not modeled.

## Results

In the following, we show example results that assess the performance of RosettaBackrub sequence tolerance predictions using three different experimental datasets that determined tolerated sequences for protein fold stability [9] and protein binding [23], [35] using phage display. Two of these tests were previously performed with an earlier Rosetta version [23] or scoring function [35]. Here we evaluate the generality of the Rosetta 3 standard protocol described in this Rosetta collection on all three datasets, compare to previous results, present a new test on a dataset of tolerated sequences for fold stability and provide an extensive set of customizable simulation and analysis tools in addition to all source code. Overall, the generalized protocol captures a significant fraction of the observed sequence space in all three datasets (Table 1), with values for the area under a ROC curve between 0.64 and 0.87, and the fraction of sequence space captured by the top 5 ranked amino acid types between 54 and 82%.

### GB1 Fold Stability Tolerated Sequence Space Prediction

The fold stability test used a dataset by Kotz et al who determined tolerated sequences for three residues in the core (L5, L7, and F30) of the B1 domain of protein G (GB1) and three residues bordering the core (T16, T18, and Y33) [9]. The authors utilized the ability of the GB1 domain to bind to the human IgG Fc domain for a phage display screen. The side chains of the six GB1 residues varied in the experiment are at least 7 Å from any heavy atom on the IgG Fc domain in the cocrystal structure between the GB1 and IgG Fc domains [47], as shown in Figure 2. Mutating the GB1 residues should thus primarily affect the stability of the GB1 domain and report on sequences tolerated for fold stability, instead of selecting sequences that modify the interaction directly. After three rounds of GB1 display on phage, using IgG as bait, the authors obtained 171 full-length GB1 sequences suitable for analysis.

The results of applying the generalized sequence tolerance prediction protocol described in Methods are shown in Figure 2. Consistent with previous studies [35], the prediction of sequence rank is often better than the absolute frequencies. Therefore, we compared the predicted ranking of the amino acid types at each position to the experimentally observed frequencies. Averaged over the six positions, 57% of the frequently observed amino acids are found in the top five predicted amino acids. This performance metric, which is helpful for gauging the usefulness of the prediction for library design or other protein engineering applications, is used along with other metrics to compare all three datasets in Table 1. For actual protein engineering applications, it is critical to correctly identify at least one “viable” (tolerated) amino acid type at each position. Here, for all six positions, the prediction finds at least one frequently observed amino acid (greater than 10% frequency) within the top five ranked amino acids. (This analysis ignores co-variation between positions, which can be obtained from analysis of the actual predicted sequences).

In this example test case, the predictions reveal bias towards the native, input sequence at five positions. Two out of those five positions, core residues L5 and F30, show the wild type sequence to be the most frequent in phage display. Two of the border positions, T16 and T18, are incorrectly biased towards the input sequence. One of those positions is flat, with no single residue having greater than 20% frequency, so it is not surprising that the input bias overwhelms the relatively weak preferences. For residue Y33, the prediction correctly ranks both frequently observed amino acids in the top five ranked amino acids and above the input wild-type tyrosine.

### Human Growth Hormone/Human Growth Hormone Receptor Interaction

The first iteration [23] of a sequence tolerance prediction method was implemented in Rosetta 2 and applied to the recapitulation of data from phage display selections of human growth hormone (hGH), using human growth hormone receptor (hGHR) as bait [14]. Besides using an entirely different implementation, which made the present computations approximately 2–20 times faster, there were several algorithmic differences between the previous approach and the generalized protocol presented here.

The main difference lies in the way sequences were scored, filtered and weighted. The earlier protocol used a scoring function parameterized for protein-protein interfaces. In addition, the score of the protein was decomposed into a “binding” score (intermolecular interactions between chains; A-B in Figure 1) and a “folding” score (intramolecular interactions, sum of A and B in Figure 1). Sequences were allowed to contribute to the calculated frequencies if their binding and folding scores fell below given cutoffs determined using the wild-type sequence scores. The generalized protocol presented here uses the Rosetta 3.2 default all-atom scoring function, including an increased histidine reference energy (see Methods), was designed to work without having a wild-type sequence, and all scores were normalized to the lowest fitness found for a given backbone. Additionally, instead of using two separate scores for weighting, a linear combination of the binding and folding scores was used. Finally, instead of using hard cutoffs, Boltzmann weighting was used to weight the contribution of a given sequence to the final position weight matrix.

**Figure 3:**
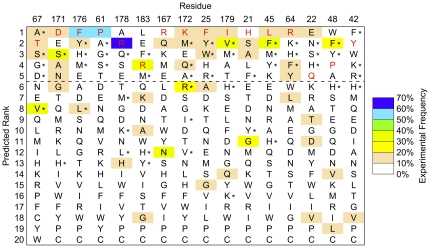
hGH/hGHR interface tolerance prediction. The generalized Rosetta 3 protocol described here was applied to rank human growth hormone (hGH) amino acids by computationally predicted frequency. The residue positions shown and their ordering are taken from previously published results using the Rosetta 2 protocol (Humphris & Kortemme, Table 2 [23]). Wild type residues, which were used in protein ensemble generation, are shown in red. For each position, an average of 59% of the amino acids observed in phage display (≥10% experimental frequency) are predicted within the top five computationally ranked amino acids (above dashed line). Overall performance was similar to previous results of the Rosetta 2 protocol. Amino acids (other than wild-type) included in the computationally selected library from the Rosetta 2 protocol are indicated with a star. If the same number of amino acids at each position is used as defined in the computational library in [23], Table 2, the Rosetta 3 protocol misses two frequently observed amino acids included by Rosetta 2 (V67 and L176). Conversely, the Rosetta 2 protocol misses three frequently observed amino acids included by Rosetta 3 (S21, A21, and E22). Both protocols share similar false positive predictions. However, the Rosetta 3 histidine reference energy reweighting (see Methods) eliminates 6 out of 8 histidine false positives (H*).

The predictions from the generalized protocol were similar to to the previous method [23] for the 16 residue positions in which a computationally selected library was described [23] (Figure 3). Using the residue-specific size of the library as previously defined (Table 2 in reference [23]), the Rosetta 3 protocol has one fewer false negative (and by definition of the fixed-size library one fewer false positive) than the Rosetta 2 protocol. These results thus highlight the transferability of the parameters and protocol used here, while providing a more general prediction framework.

### PDZ/Peptide Interaction

**Figure 4:**
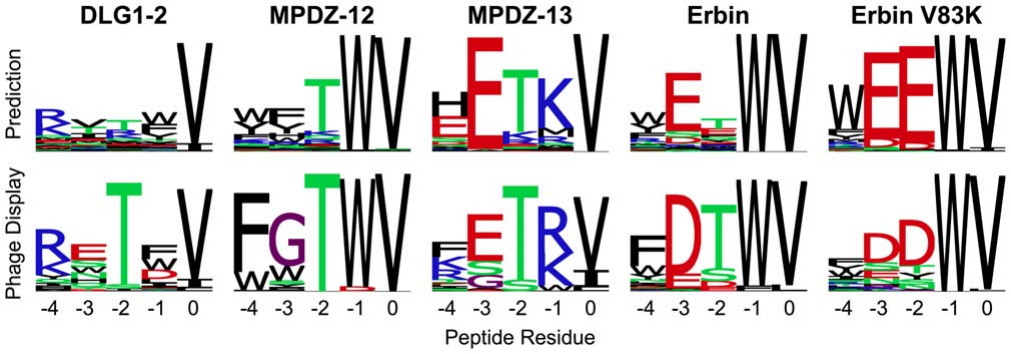
PDZ/peptide interface tolerance predictions. Shown are 5 representative examples of predictions with the generalized protocol, compared to experimental data from phage display. The Erbin V83K interface prediction involved making the indicated point mutant (V83K) to the PDZ domain prior to backrub ensemble generation (an example of a “premutated” position).

The third test dataset contains peptide sequences selected by phage display to bind to PDZ domains [15]. To determine if the generalized protocol and scripts described here produce similar results to those previously published on the PDZ-peptide dataset [35], we performed 5 representative PDZ/peptide interface specificity predictions. (For details on methodological differences between the published and current protocols, see the Methods section.) Computational and experimental sequence logos are shown in Figure 4. The correspondence to experiment is overall similar to the previous protocol [35], with the largest difference observed in the absolute frequency of amino acids, as shown in Table 1. The primary changes are reductions in the preferences for R/K at position −4 and T at position −2 for the DLG1-2 PDZ domain, as well as the preference for T at position −2 for the Erbin PDZ domain. These differences likely come from the restoration of environment dependent hydrogen bonds in the current protocol, which weakens hydrogen bonds in solvent exposed areas.

### Sampling Efficiency and Boltzmann Factors

**Figure 5:**
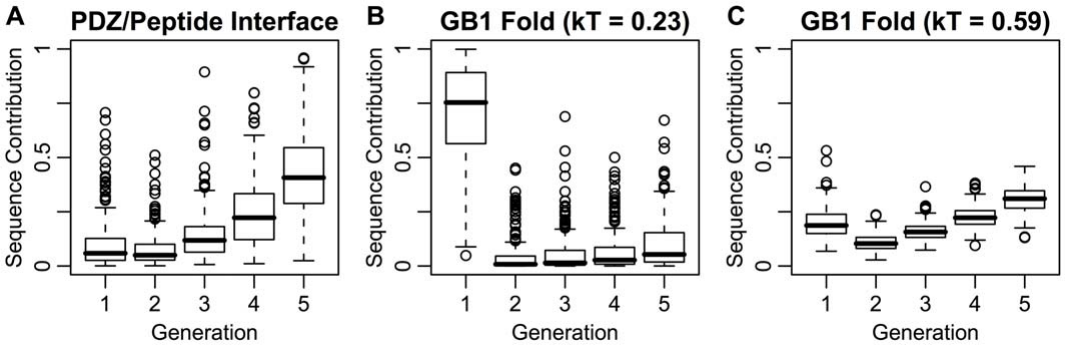
Sequences from later genetic algorithm generations contribute more in interface design prediction than in protein stability design prediction. The total Boltzmann weights in the final PWM for the new sequences sampled in each generation were calculated. The distribution of contributions for each generation across the 200 simulations (one simulation for each backbone in the backrub ensemble) is shown. Boxes span from the first quartile to the third quartile, with the line indicating the median. Whiskers extend to the most extreme data point within 1.5 times the interquartile range of the box. Circles show data points beyond that limit. **A.** Because the fitness function used for protein-protein interfaces (here shown for a complex between the second PDZ domain of DLG1 and peptides) is different from the fitness function used for optimization of side chain packing, the genetic algorithm is important for enriching the population in sequences predicted to be better binders. **B.** For optimization of protein fold stability (designing positions in the GB1 core), the initial full protein design phase is very effective at finding a low energy sequence, which dominates the contribution to the position weight matrix (PWM) when the same Boltzmann factor (*kT* = 0.23) is used. **C.** When the Boltzmann factor is optimized to minimize the average absolute difference between experiment and computation (*kT* = 0.59), the contribution of the later generations increases significantly.

From an algorithmic point of view, one of the primary differences between the protocols presented here for interface vs. fold stabilization is whether the fitness function is reweighted (interfaces) or not reweighted (fold stabilization) after side chain packing. The first generation of the genetic algorithm consists of random sequences as well as the sequence with the best raw score as defined by the non-reweighted fitness function. Because the reweighting changes the fitness function, this optimized sequence often does not score as well relative to sequences that evolve in later generations in the case of interface stabilization. This leads to a lower overall contribution of the first generation sequences to the final PWM (Figure 5A). However, the reweighted fitness quickly improve, leading to a median fifth generation PWM contribution of 40%.

By contrast, when optimizing sequences to preserve fold stability, the raw Rosetta score for optimization of intramolecular side chain packing and reweighted Rosetta fitness score for Boltzmann weighting are identical. Using the same Boltzmann factor as for interface prediction, the first generation overwhelmingly dominates the contribution to the final PWM (Figure 5B). The primary contribution of the first generation comes from the sequence that showed the best overall side chain packing. It typically takes several generations for new sequences to be discovered that score close enough to that sequence to make a significant contribution to the PWM. This imbalance may be partially an artifact of the Boltzmann factor that was not previously assessed for prediction of tolerated sequences for fold stability. The Boltzmann factor increases from 0.23 (taken from the PDZ-peptide study) to 0.59 if it is reoptimized to produce the highest similarity between the predicted and experimental PWMs (Figure 5C). Here, the contributions of the different generations are more balanced. Of note, this change in Boltzmann factor does not significantly change the sequence ranks (data not shown), but does make the computational predictions match the relative flatness of the experimental PWM better. If this protocol is applied to other monomeric systems where absolute frequencies matter, the Boltzmann factor of 0.59 may provide a more useful starting point.

Another algorithmic consideration is the influence of introducing backbone flexibility into the prediction method. To determine the effect backbone flexibility had in our simulations, we repeated the predictions without backrub moves and computed overall performance (Table S1). The results with the heterogeneous test set used here mirror the previous finding for PDZ-peptide interactions [35], namely that backbone flexibility improves predictions by most metrics. The only place where the fixed backbone method showed better performance was the Fraction Top 5 scores for the GB1 dataset. Overall prediction performance improved with an incerasing number of backbones until convergence was reached at about 20 backbones (Figure S2) for the three datasets tested here.

A final point of comparison can be made to a naïve model, in which residues with similar chemical properties to those in the input structure are given equal weight in a predicted PWM. Using the unmodified *kT* of 0.23, the prediction method presented here also outperforms the naïve model by most performance metrics (Table S2).

### Discussion

One of the key assumptions made in the method described here is that the backbone structures generated with the input sequence will adequately sample backbones that will accommodate other amino acid sequences. While we have shown here and in previous work that incorporation of backbone flexibility improves prediction of tolerated sequence space [23], [35], side chain order parameters [32], and residual dipolar couplings [24], this and previous studies indicate that there are limitations to that assumption. To adequately sample both backbone and sequence space, variants of simultaneous or iterative sampling strategies [27], [28] are likely necessary. We have made initial attempts at adding iteration to this method and others, but found that the simulations end up trapped in local minima of sequence space, with the backbones retaining the bias towards the sequence that they start with. Often, the solution to limited sampling is to increase the simulation temperature, which can be done when the backbone is fixed. However, when the backbone is flexible, increasing the temperature can lead to protein unfolding and sampling of unproductive regions of sequence space. Application of constraints, restraints, or other sampling methods may be required to overcome that problem.

While the uses of this protocol to date have been limited to protein-protein interfaces and monomeric protein folds, there are several other applications that it can also be generalized to. For instance, this method could be leveraged in prediction of the amino acid sequences that will bind to a small molecule substrate, cofactor, or inhibitor, as well as for protein-DNA and protein-RNA interfaces. Another potential application would be stabilizing particular conformations of loops or domains. For that purpose, one could place the backbone into a preferred conformation at the outset, and then upweight the interaction energies between the residues that are desired to interact. While many design problems can be described using a single state, adaptation of the code described here could be used to generate a set of sequences that satisfy multiple states or constraints [48]–[50].

## Supporting Information

Figure S1 **Increasing the number of backbones reduces stochastic variation.** 2000 backbones were generated for each of the prediction simulations used here, resulting in approximately 240 million sequence scores. The frequencies calculated from the entire dataset (*kT* = 0.23) were treated as the ground truth and used to calculate the root mean squared error (RMSE) for subsets of the data using 200 (red), 100 (orange), 50 (cyan), and 20 (purple) backbones each. A. Frequency data were divided into 20 equally spaced bins and the predicted frequency RMSE was calculated for each bin. For example, if the method is applied using 100 backbones, and an amino acid frequency is predicted to be 0.425, then the estimated error is approximately 0.125 (dashed lines). B. The data were divided by rank and the predicted rank RMSE was calculated for each rank. For example, if this method is applied using 20 backbones, and an amino acid rank is predicted to be 3, then the estimated error is approximately 1.9 (dashed lines). For 20 backbones, the stochastic contribution to the root mean squared error (RMSE) of the predicted frequency can be up to 0.25, which is 25% of the dynamic range. The predicted ranks are more robust, with an RMSE of up to 2.5, or 12.5% of the dynamic range. 100 and 200 backbones reduce the stochastic error by approximately 2-fold and 2.5-fold over 20 backbones.
(TIFF)

Figure S2 **Dependence of prediction performance on number of backbones.** Distributions of area under ROC curve (AUC) values are shown for varying numbers of backbones. Prediction performance plateaus at approximately 20 backbones. Each boxplot shows the distribution of mean AUC values for 50 sets of independent backbones (mean AUC values were computed across all datasets, from the equivalent of rows 1, 4, and 5 of Table 1). Horizontal lines represent the median, the box spans the interquartile range (IQR), whiskers extend to the furthest data point up to 1.5 times the IQR from the box, and data points outside the range are shown with circles. This figure used the same data that were generated for Figure S1).
(TIFF)

Figure S3 **Sequence tolerance prediction for the hGH/hGHR interface is not highly sensitive to data processing parameters.** For the 35 designed positions in the human growth hormone (hGH)/human growth hormone receptor (hGHR), position weight matrices (PWM) were generated using a grid of intramolecular weights and percentile cutoffs. **A.** At each grid point, the value of *kT* was fit such that the average number of bits of information matched that observed in phage display (i.e. 0.89 bits, see Table 1). **B.** In the resulting PWMs, the average absolute difference (AAD) between phage display and prediction shows little sensitivity to the processing parameters. The point with parameters equivalent to those found in the PDZ/peptide predictions (0.4 intramolecular weight, 0.5 percentile) is only slightly worse (by 0.04% AAD) than the lowest (best) AAD sampled on the grid. The other rank-based metrics also do not change significantly across the same parameter space and are less sensitive to changes in *kT* (data not shown).
(TIFF)

Figure S4 **hGH/hGHR interface tolerance prediction for all residues.** Human growth hormone (hGH) amino acids are ranked by computationally predicted frequency using the generalized Rosetta 3 protocol described here. Wild type residues, which were used in protein ensemble generation, are shown in red. (Representation and color coding is as shown in Figure 3 in the main text).
(TIFF)

Table S1 **Summary of fixed backbone prediction performance.** As a fraction of the dynamic range of the performance metrics, the predicted bits of information, AAD, AUC, and Rank Top metrics (averaged over all datasets) are better with backrub sampling (see Table 1) by 9.4%, 9.1%, 1.6%, and 1.1%, respectively. The only performance metric that was better (by 3.8%) without backrub sampling was Fraction Top 5. This improvement came primarily from the GB1 dataset. Fraction Top 5 was found to be the most variable performance metric across replicated predictions (Table 1).
(PDF)

Table S2 **Summary of naïve model prediction performance.** Naïve predictions were constructed by generating position weight matrices in which the PDB amino acid and amino acids in its similarity group were given equal weight, and all other amino acids given zero weight. The similarity groups were as follows: DENQ, RKH, LIVM, FYW, PAG, ST, and C [23]. All metrics for the performance of the naïve model (Fraction Top 5, AAD, AUC and Rank Top) were worse than those shown in Table 1, with the exception of the hGH/hGHR AAD for the 16-residue set. In addition to performing better than a naïve model, the method described in the main text also does better than random, as evidenced by the area under ROC curves (AUC) being greater than random (0.5) for all datasets (Table 1).
(PDF)

Text S1 **Background on the “standard” and “score12” Rosetta energy function weights**
(PDF)

Dataset S1 **Protocol Capture**
(BZ2)
