# Supplementary figures and images for: Predicting the Tolerated Sequences for Proteins and Protein Interfaces Using RosettaBackrub Flexible Backbone Design
Source: PLoS One. 2011 Jul 18;6(7):e20451. doi: 10.1371/journal.pone.0020451 (PMC3138746; doi:10.1371/journal.pone.0020451)

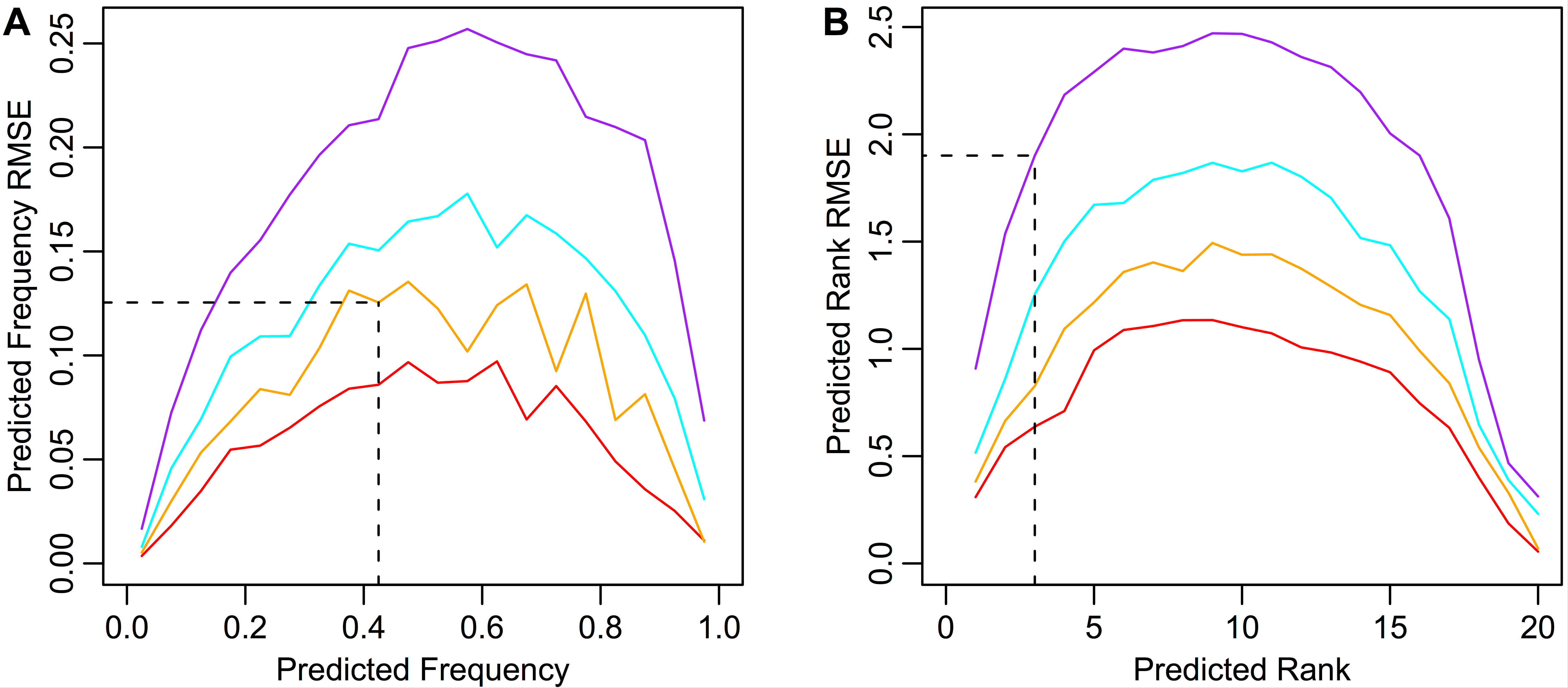

Supplement: Figure S1 — Increasing the number of backbones reduces stochastic variation. 2000 backbones were generated for each of the prediction simulations used here, resulting in approximately 240 million sequence scores. The frequencies calculated from the entire dataset (kT = 0.23) were treated as the ground truth and used to calculate the root mean squared error (RMSE) for subsets of the data using 200 (red), 100 (orange), 50 (cyan), and 20 (purple) backbones each. A. Frequency data were divided into 20 equally spaced bins and the predicted frequency RMSE was calculated for each bin. For example, if the method is applied using 100 backbones, and an amino acid frequency is predicted to be 0.425, then the estimated error is approximately 0.125 (dashed lines). B. The data were divided by rank and the predicted rank RMSE was calculated for each rank. For example, if this method is applied using 20 backbones, and an amino acid rank is predicted to be 3, then the estimated error is approximately 1.9 (dashed lines). For 20 backbones, the stochastic contribution to the root mean squared error (RMSE) of the predicted frequency can be up to 0.25, which is 25% of the dynamic range. The predicted ranks are more robust, with an RMSE of up to 2.5, or 12.5% of the dynamic range. 100 and 200 backbones reduce the stochastic error by approximately 2-fold and 2.5-fold over 20 backbones. (TIFF) [file pone.0020451.s001.tiff]

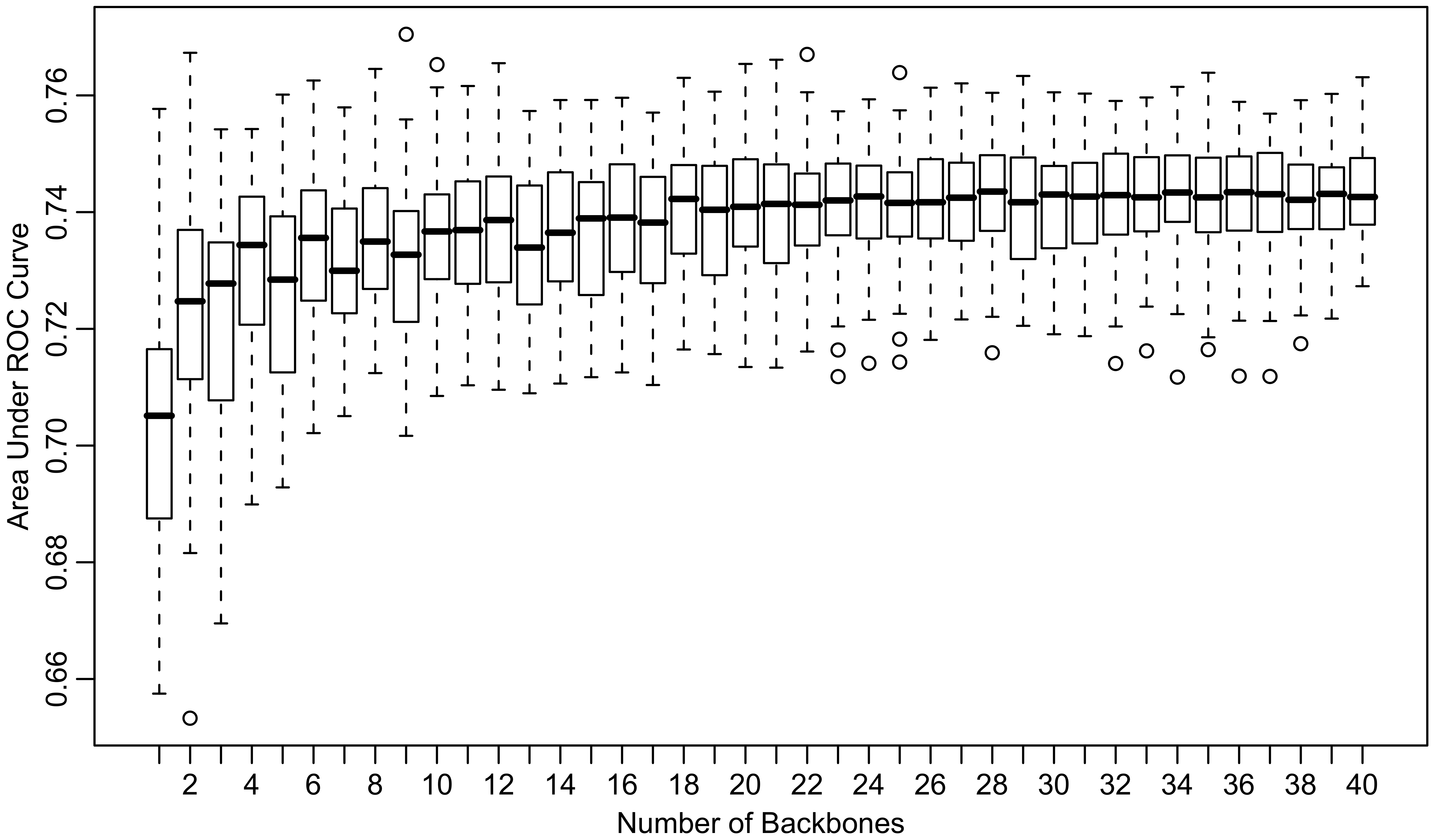

Supplement: Figure S2 — Dependence of prediction performance on number of backbones. Distributions of area under ROC curve (AUC) values are shown for varying numbers of backbones. Prediction performance plateaus at approximately 20 backbones. Each boxplot shows the distribution of mean AUC values for 50 sets of independent backbones (mean AUC values were computed across all datasets, from the equivalent of rows 1, 4, and 5 of Table 1). Horizontal lines represent the median, the box spans the interquartile range (IQR), whiskers extend to the furthest data point up to 1.5 times the IQR from the box, and data points outside the range are shown with circles. This figure used the same data that were generated for Figure S1). (TIFF) [file pone.0020451.s002.tiff]

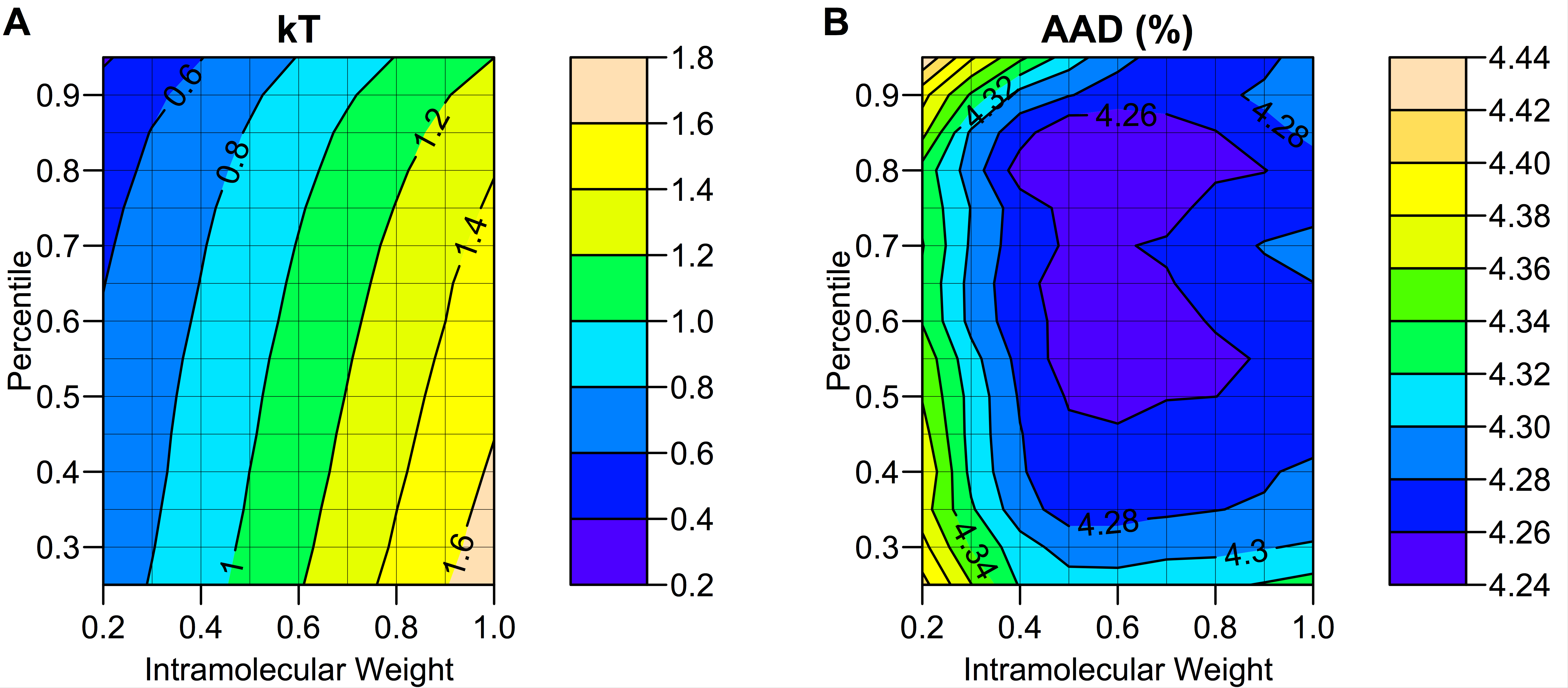

Supplement: Figure S3 — Sequence tolerance prediction for the hGH/hGHR interface is not highly sensitive to data processing parameters. For the 35 designed positions in the human growth hormone (hGH)/human growth hormone receptor (hGHR), position weight matrices (PWM) were generated using a grid of intramolecular weights and percentile cutoffs. A. At each grid point, the value of kT was fit such that the average number of bits of information matched that observed in phage display (i.e. 0.89 bits, see Table 1). B. In the resulting PWMs, the average absolute difference (AAD) between phage display and prediction shows little sensitivity to the processing parameters. The point with parameters equivalent to those found in the PDZ/peptide predictions (0.4 intramolecular weight, 0.5 percentile) is only slightly worse (by 0.04% AAD) than the lowest (best) AAD sampled on the grid. The other rank-based metrics also do not change significantly across the same parameter space and are less sensitive to changes in kT (data not shown). (TIFF) [file pone.0020451.s003.tiff]

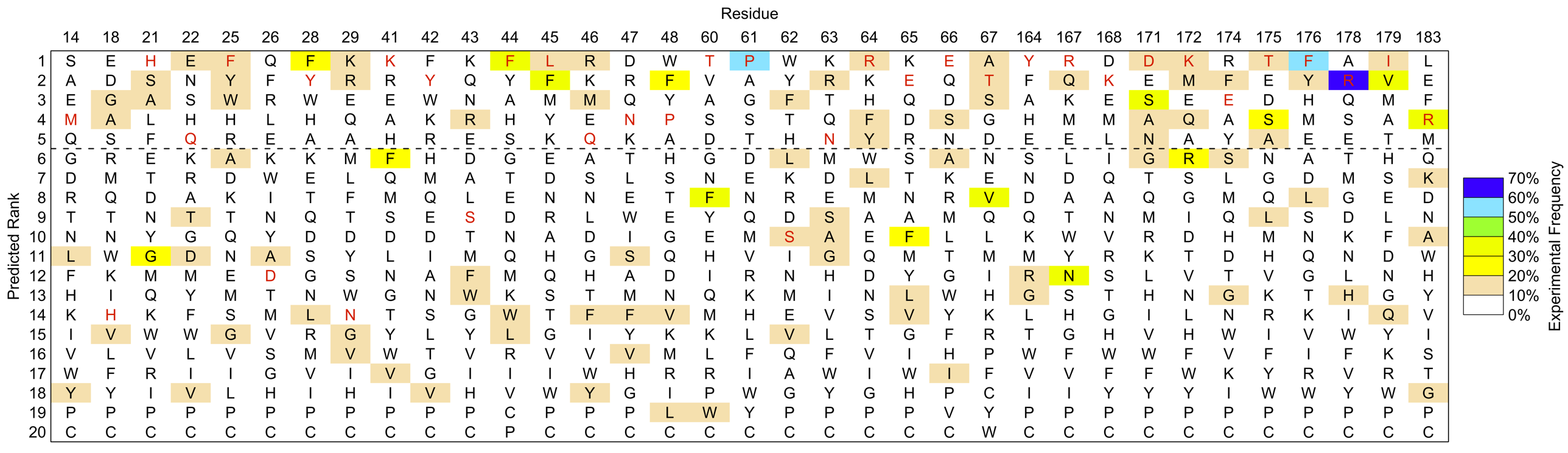

Supplement: Figure S4 — hGH/hGHR interface tolerance prediction for all residues. Human growth hormone (hGH) amino acids are ranked by computationally predicted frequency using the generalized Rosetta 3 protocol described here. Wild type residues, which were used in protein ensemble generation, are shown in red. (Representation and color coding is as shown in Figure 3 in the main text). (TIFF) [file pone.0020451.s004.tiff]

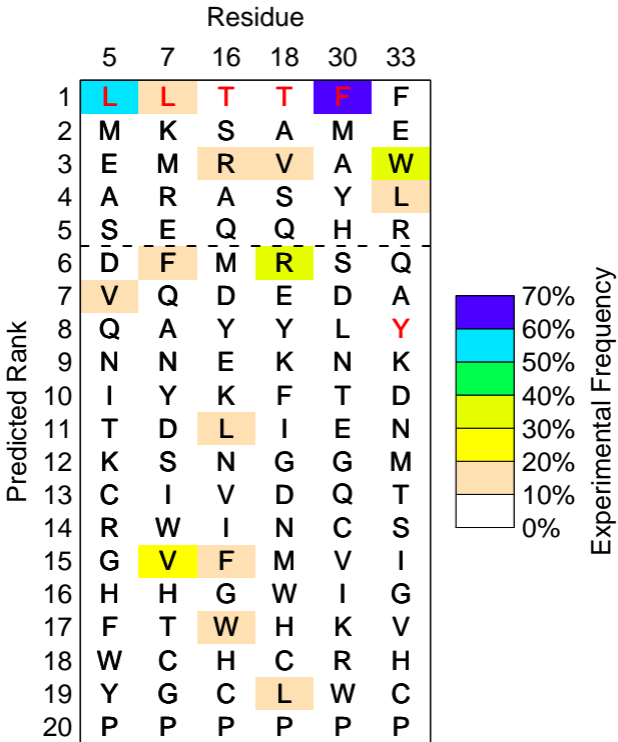

Supplement: Dataset S1 — Protocol Capture (BZ2) [file pone.0020451.s008.bz2 › DatasetS1/output_files/Figure2_2QMTSeqRank.pdf]

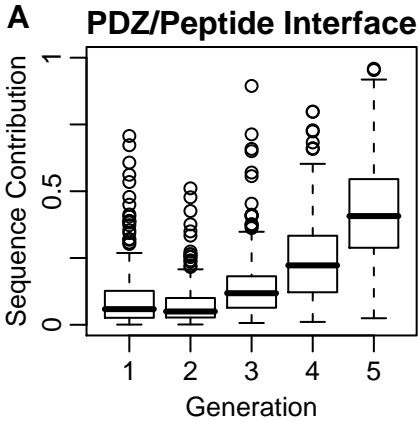

Supplement: Dataset S1 — Protocol Capture (BZ2) [file pone.0020451.s008.bz2 › DatasetS1/output_files/Figure5_2I0LGenContrib.pdf]

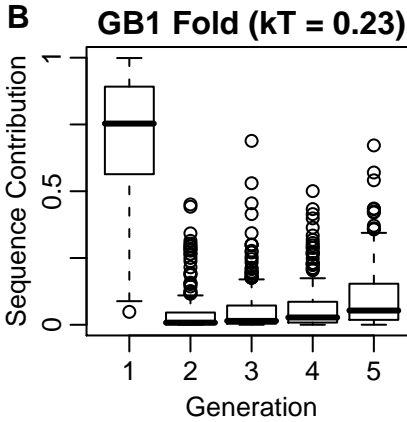

Supplement: Dataset S1 — Protocol Capture (BZ2) [file pone.0020451.s008.bz2 › DatasetS1/output_files/Figure5_2QMTGenContrib.pdf]

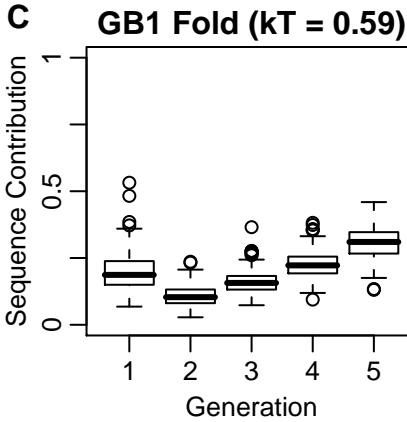

Supplement: Dataset S1 — Protocol Capture (BZ2) [file pone.0020451.s008.bz2 › DatasetS1/output_files/Figure5_2QMTGenContrib_Opt.pdf]

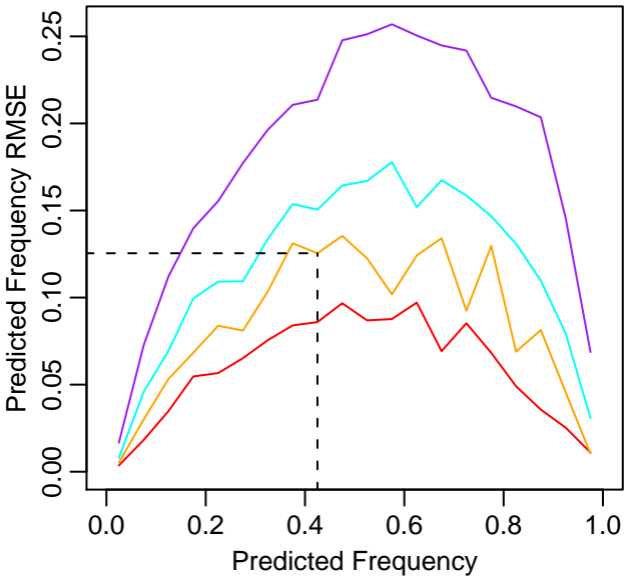

Supplement: Dataset S1 — Protocol Capture (BZ2) [file pone.0020451.s008.bz2 › DatasetS1/output_files/FigureS1_FreqRMSE.pdf]

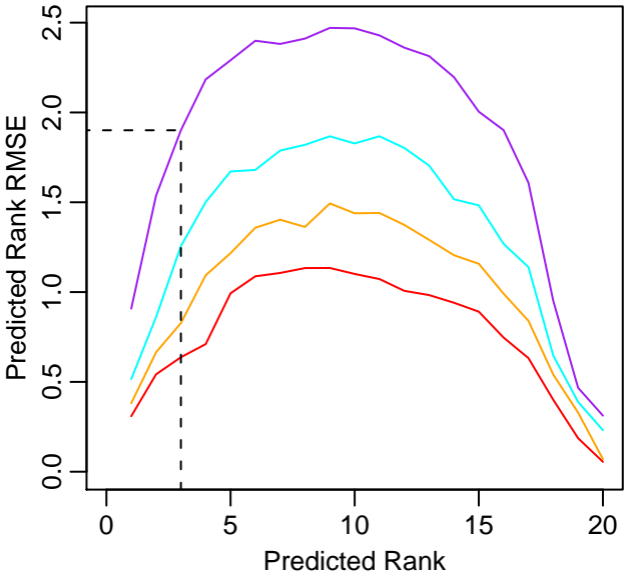

Supplement: Dataset S1 — Protocol Capture (BZ2) [file pone.0020451.s008.bz2 › DatasetS1/output_files/FigureS1_RankRMSE.pdf]

# AAD (%)

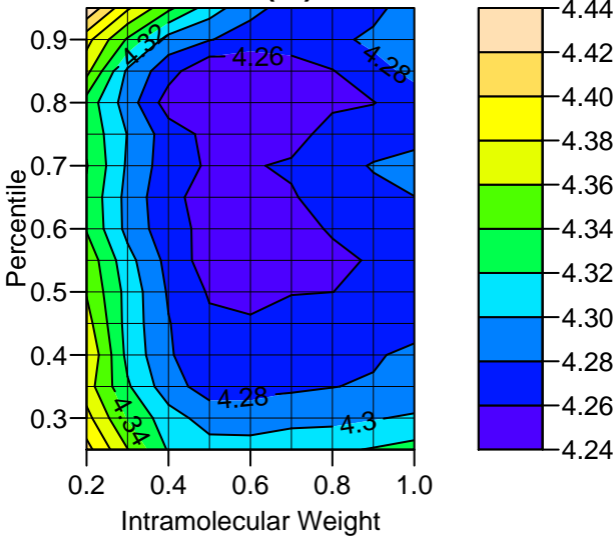

Supplement: Dataset S1 — Protocol Capture (BZ2) [file pone.0020451.s008.bz2 › DatasetS1/output_files/FigureS3_AADArray.pdf]

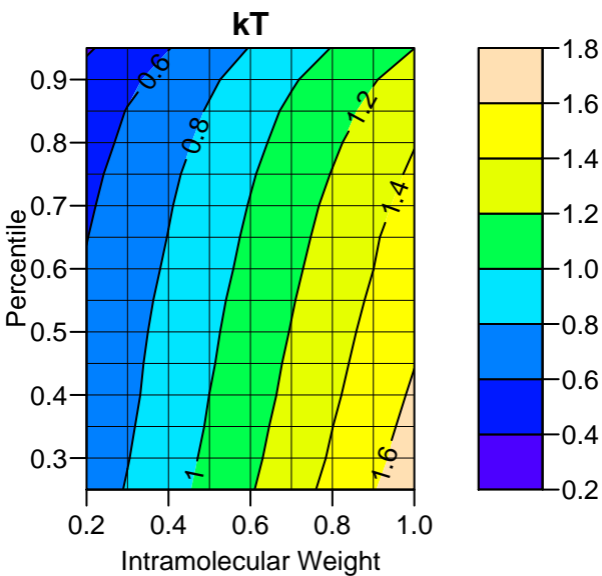

Supplement: Dataset S1 — Protocol Capture (BZ2) [file pone.0020451.s008.bz2 › DatasetS1/output_files/FigureS3_kTArray.pdf]
