# Supplementary material for: Predicting the Tolerated Sequences for Proteins and Protein Interfaces Using RosettaBackrub Flexible Backbone Design
Source: PLoS One. 2011 Jul 18;6(7):e20451. doi: 10.1371/journal.pone.0020451 (PMC3138746; doi:10.1371/journal.pone.0020451)
Supplement: Table S1 — Summary of fixed backbone prediction performance. As a fraction of the dynamic range of the performance metrics, the predicted bits of information, AAD, AUC, and Rank Top metrics (averaged over all datasets) are better with backrub sampling (see Table 1) by 9.4%, 9.1%, 1.6%, and 1.1%, respectively. The only performance metric that was better (by 3.8%) without backrub sampling was Fraction Top 5. This improvement came primarily from the GB1 dataset. Fraction Top 5 was found to be the most variable performance metric across replicated predictions (Table 1). (PDF) [file pone.0020451.s005.pdf]

	Proteins	Residue positions	Bits of information		Fraction Top 5 (%)	AAD (%)	AUC	Rank Top
			Phage display	Predicted				
GB1 (*kT*=0.23)	1	6	1.58	4.25	70.8	7.77	0.73	6.33
GB1 (*kT*=0.66)	1	6	1.58	1.00	76.4	4.53	0.75	6.33
hGH/hGHR[1]	1	16	1.19	3.69	52.8	7.51	0.68	7.38
hGH/hGHR[2]	1	35	0.89	3.56	42.0	7.81	0.62	7.86
PDZ/Peptide	5	25	3.11	2.82	81.0	5.61	0.84	3.36

[1] 16 designed hGH amino acid positions as defined in [23] and shown in Figure 3.

[2] All designed hGH amino acid positions shown in Figure S4.
