# Supplementary material for: Predicting the Tolerated Sequences for Proteins and Protein Interfaces Using RosettaBackrub Flexible Backbone Design
Source: PLoS One. 2011 Jul 18;6(7):e20451. doi: 10.1371/journal.pone.0020451 (PMC3138746; doi:10.1371/journal.pone.0020451)
Supplement: Table S2 — Summary of naïve model prediction performance. Naïve predictions were constructed by generating position weight matrices in which the PDB amino acid and amino acids in its similarity group were given equal weight, and all other amino acids given zero weight. The similarity groups were as follows: DENQ, RKH, LIVM, FYW, PAG, ST, and C [23]. All metrics for the performance of the naïve model (Fraction Top 5, AAD, AUC and Rank Top) were worse than those shown in Table 1, with the exception of the hGH/hGHR AAD for the 16-residue set. In addition to performing better than a naïve model, the method described in the main text also does better than random, as evidenced by the area under ROC curves (AUC) being greater than random (0.5) for all datasets (Table 1). (PDF) [file pone.0020451.s006.pdf]

	Proteins	Residue positions	Bits of information		Fraction Top 5 (%)[3]	AAD (%)	AUC	Rank Top
			Phage display	Predicted				
GB1	1	6	1.58	2.79	52.8	6.39	0.71	9.00
hGH/hGHR[1]	1	16	1.19	2.67	44.3	6.71	0.67	12.63
hGH/hGHR[2]	1	35	0.89	2.67	27.9	7.52	0.57	14.52
PDZ/Peptide	5	25	3.11	2.72	68.7	6.61	0.79	7.96

[1]16 designed hGH amino acid positions as defined in [23] and shown in Figure 3.

[2]All designed hGH amino acid positions shown in Figure S4.

[3]Naïve predictions, which rank up to 4 amino acids, do artificially poorly with Fraction Top 5.
