# Supplementary material for: Predicting the Tolerated Sequences for Proteins and Protein Interfaces Using RosettaBackrub Flexible Backbone Design
Source: PLoS One. 2011 Jul 18;6(7):e20451. doi: 10.1371/journal.pone.0020451 (PMC3138746; doi:10.1371/journal.pone.0020451)
Supplement: Text S1 — Background on the “standard” and “score12” Rosetta energy function weights (PDF) [file pone.0020451.s007.pdf]

Background on the “standard” and “score12” Rosetta Energy Function Weights

In Rosetta 2, side chain optimization via simulated annealing was done with weights (called “packer” weights) now defined as “standard” in Rosetta 3. Full backbone optimization in Rosetta 2 differed from these packer weights by including several small changes. First, short-range (i.e. alpha helical) hydrogen bond weights were halved. Second, a scoring term based on smoothed Ramachandran plots was added to better restrain phi/psi angles. To compensate for the addition of the Ramachandran score, the weight was halved for the score term taking into account the probability of an amino acid given phi and psi. In Rosetta 3, these three changes were incorporated into the “score12 patch”. Rosetta 3 also added an energy term restraining the omega peptide bond angle to the default scoring function, which was similarly incorporated into the score12 patch. Rosetta 3 now generally uses the same scoring function for side chain optimization and full backbone relaxation, which is “score12”, including the changes included in the “score 12 patch”, by default.
