# Supplementary material for: Predicting the Tolerated Sequences for Proteins and Protein Interfaces Using RosettaBackrub Flexible Backbone Design
Source: PLoS One. 2011 Jul 18;6(7):e20451. doi: 10.1371/journal.pone.0020451 (PMC3138746; doi:10.1371/journal.pone.0020451)
Supplement: Dataset S1 — Protocol Capture (BZ2) [file pone.0020451.s008.bz2 › DatasetS1/output_files/2QMT/specificity_boxplot.pdf]

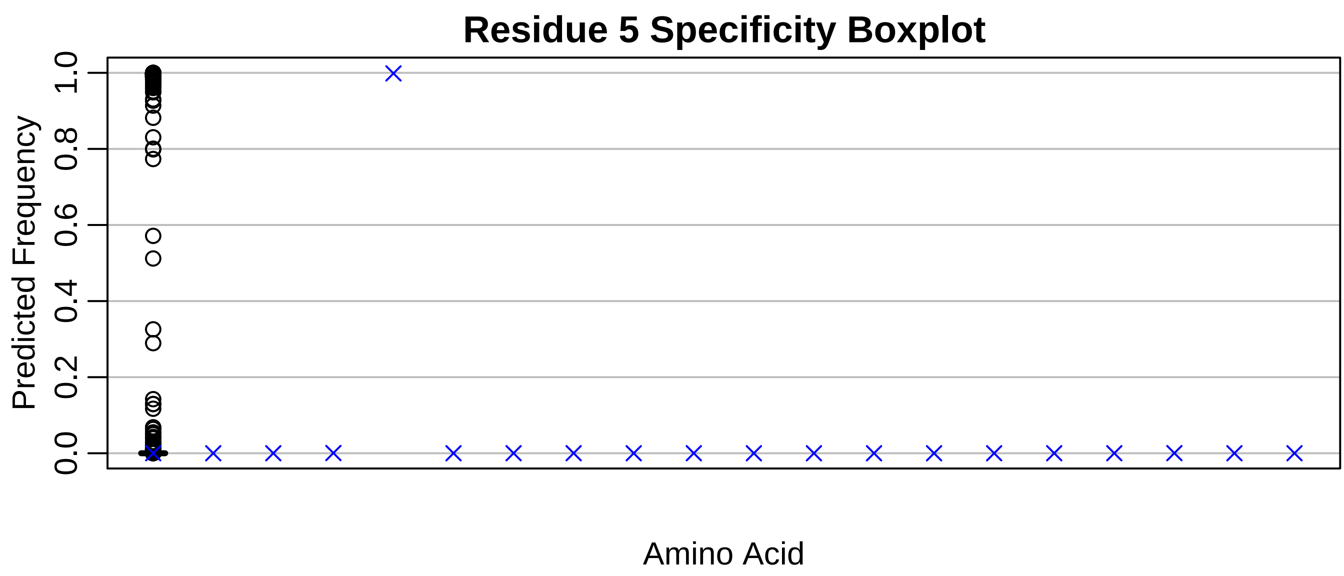

Residue 5 Specificity Boxplot
Predicted Frequency
0.0
0.2
0.4
0.6
0.8
1.0
Amino Acid

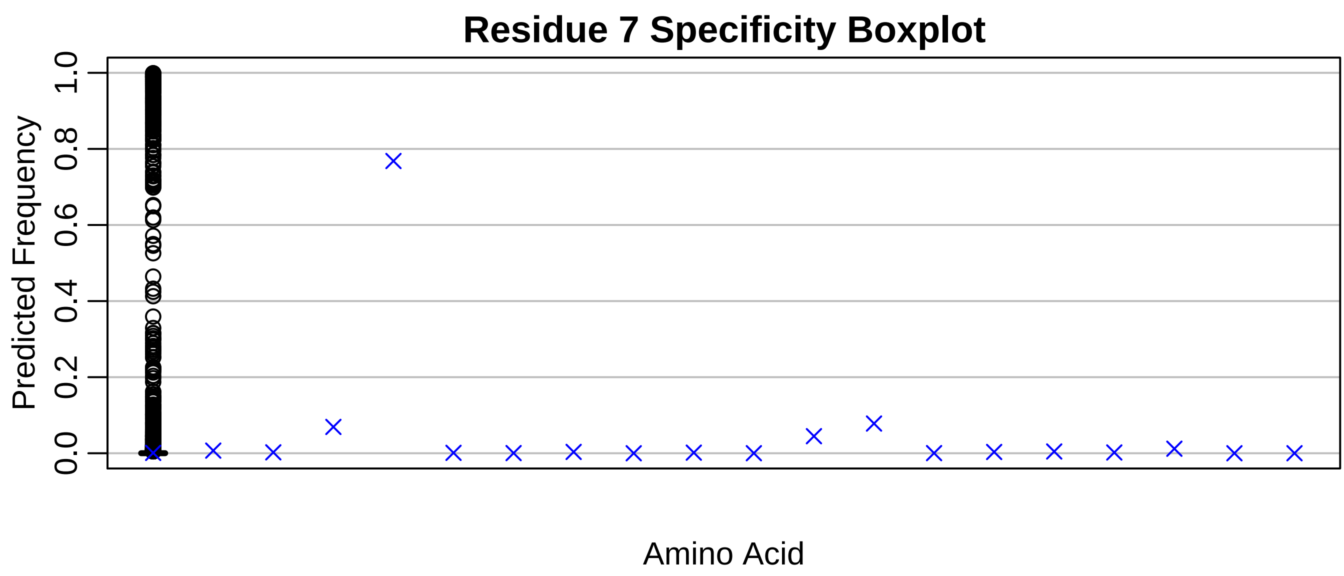

Residue 7 Specificity Boxplot
Predicted Frequency
1.0
0.8
0.6
0.4
0.2
0.0
Amino Acid

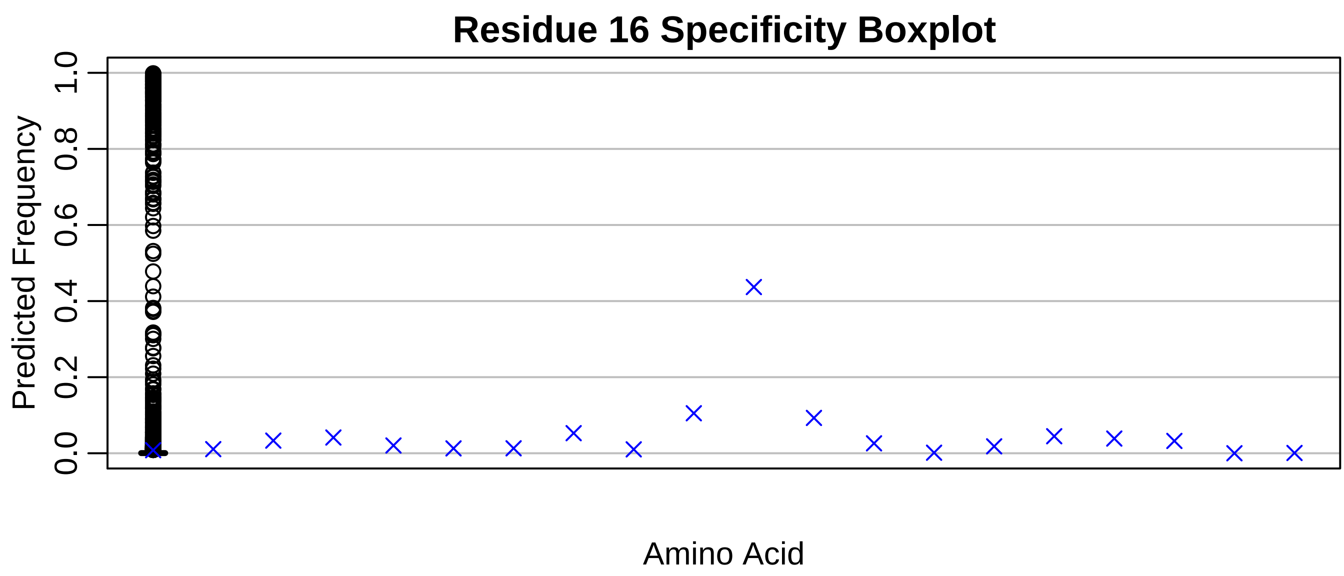
Residue 16 Specificity Boxplot
Predicted Frequency
0.0
0.2
0.4
0.6
0.8
1.0
Amino Acid

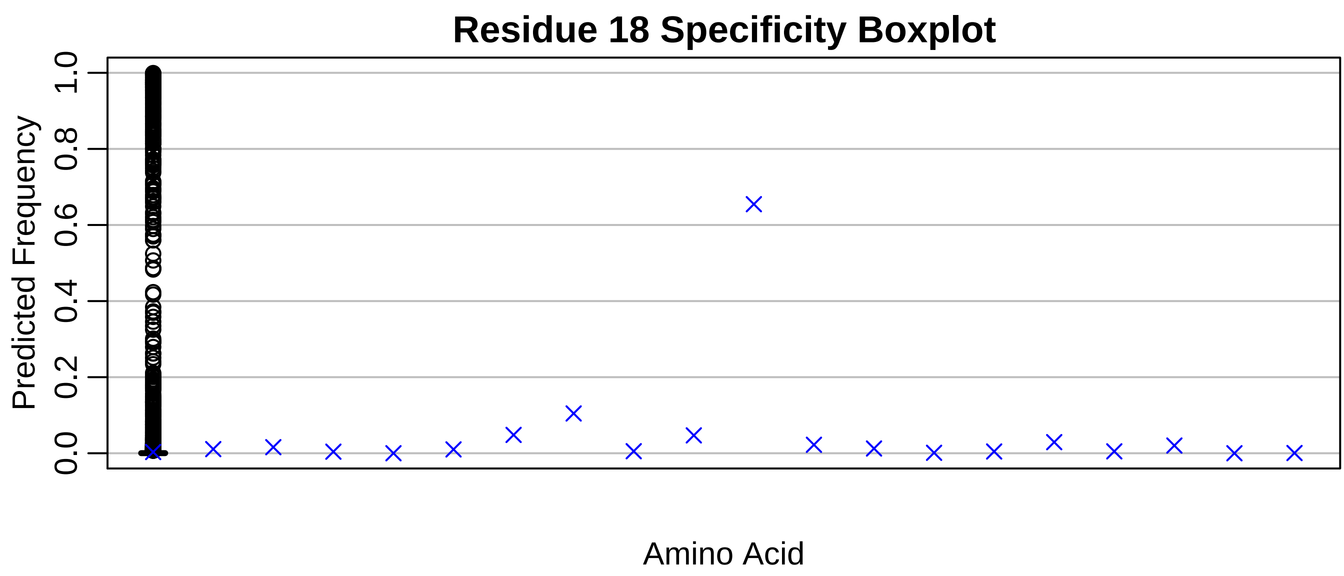

Residue 18 Specificity Boxplot
Predicted Frequency
0.0
0.2
0.4
0.6
0.8
1.0
Amino Acid

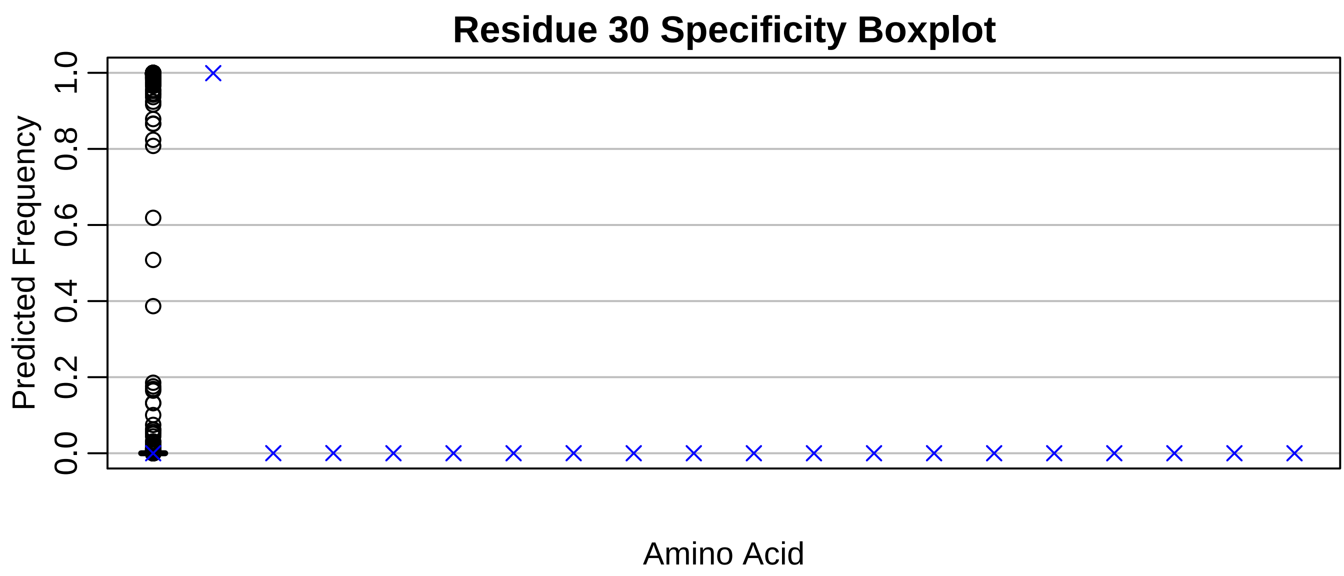
Residue 30 Specificity Boxplot
Predicted Frequency
1.0
0.8
0.6
0.4
0.2
0.0
Amino Acid

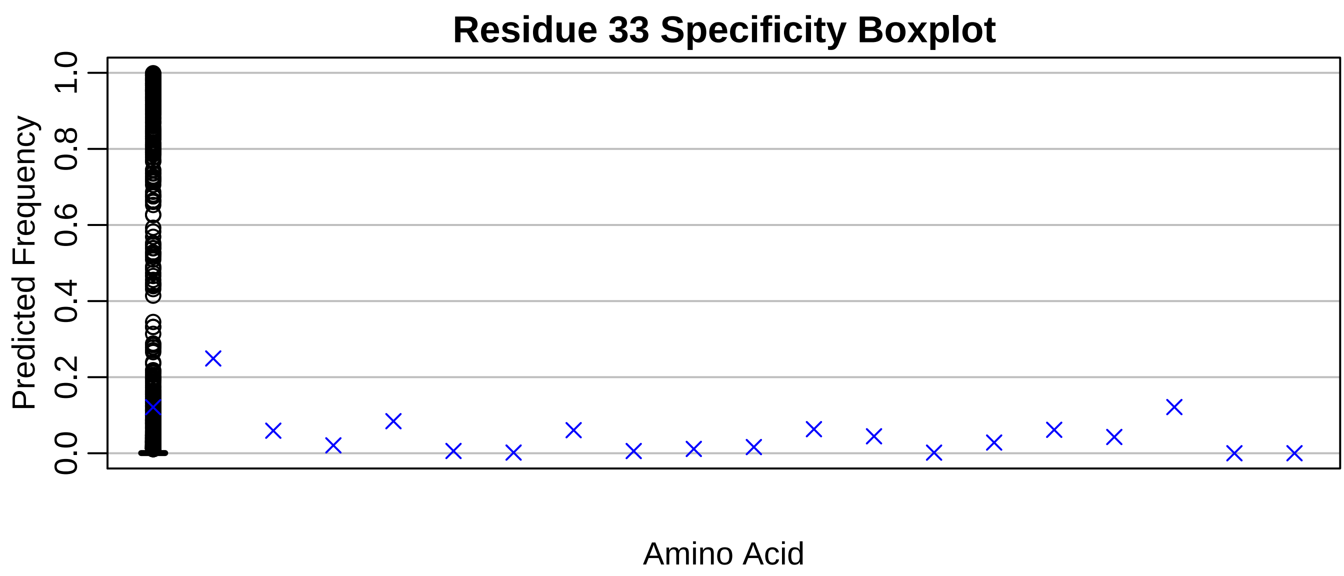

Residue 33 Specificity Boxplot
Predicted Frequency
0.0
0.2
0.4
0.6
0.8
1.0
Amino Acid
