# Supplementary material for: Predicting the Tolerated Sequences for Proteins and Protein Interfaces Using RosettaBackrub Flexible Backbone Design
Source: PLoS One. 2011 Jul 18;6(7):e20451. doi: 10.1371/journal.pone.0020451 (PMC3138746; doi:10.1371/journal.pone.0020451)
Supplement: Dataset S1 — Protocol Capture (BZ2) [file pone.0020451.s008.bz2 › DatasetS1/output_files/2I0L_A_C_V2006/specificity_boxplot.pdf]

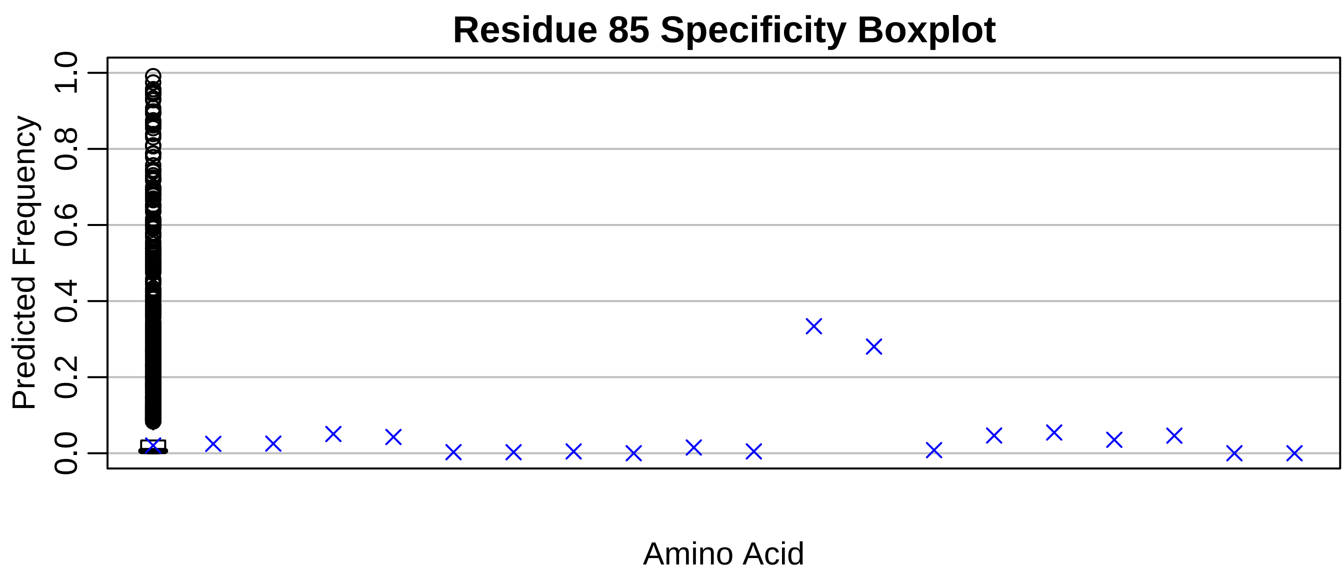
Residue 85 Specificity Boxplot
Predicted Frequency
0.0
0.2
0.4
0.6
0.8
1.0
Amino Acid

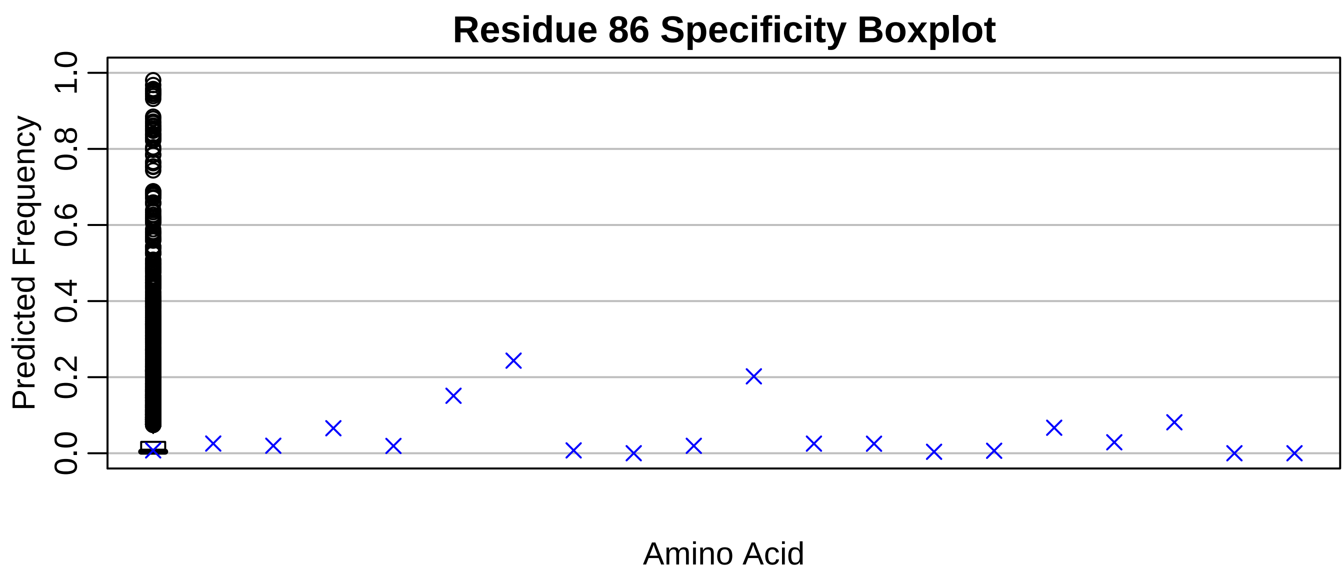

Residue 86 Specificity Boxplot
Predicted Frequency
0.0
0.2
0.4
0.6
0.8
1.0
Amino Acid

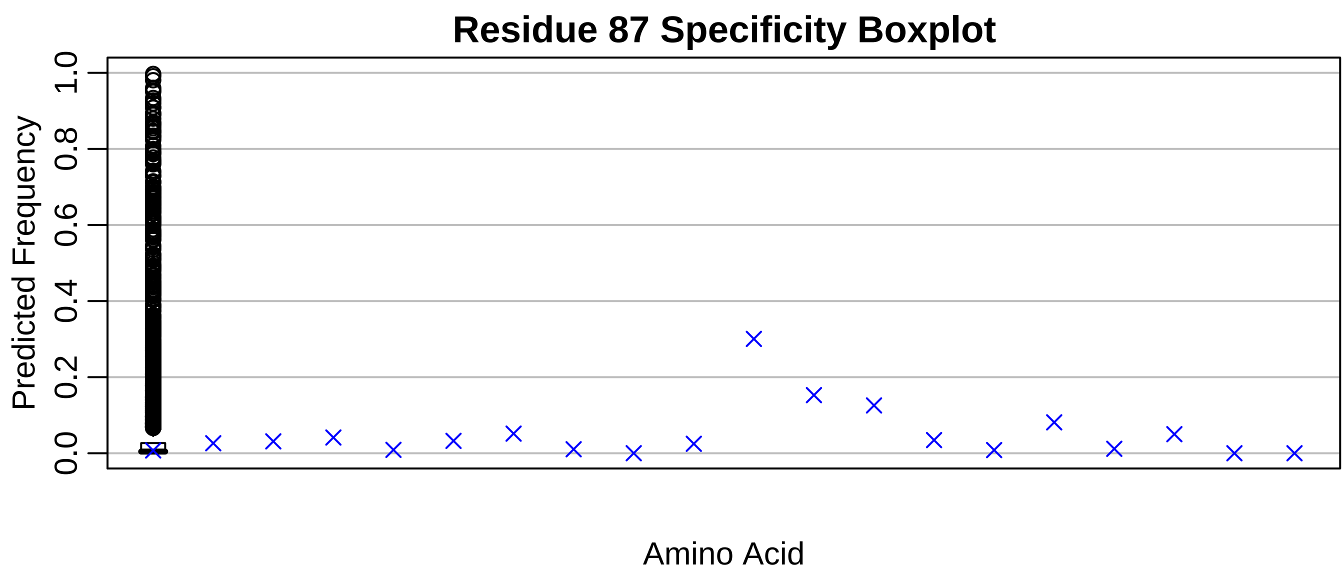

Residue 87 Specificity Boxplot
Predicted Frequency
0.0
0.2
0.4
0.6
0.8
1.0
Amino Acid

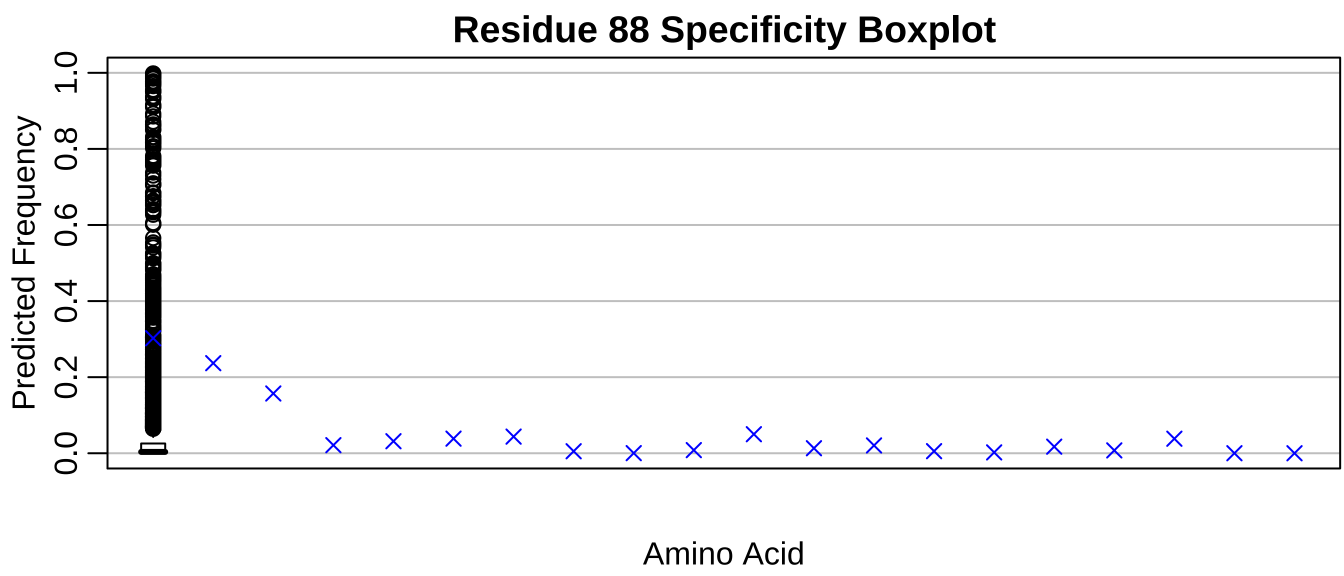
Residue 88 Specificity Boxplot
Predicted Frequency
0.0
0.2
0.4
0.6
0.8
1.0
Amino Acid

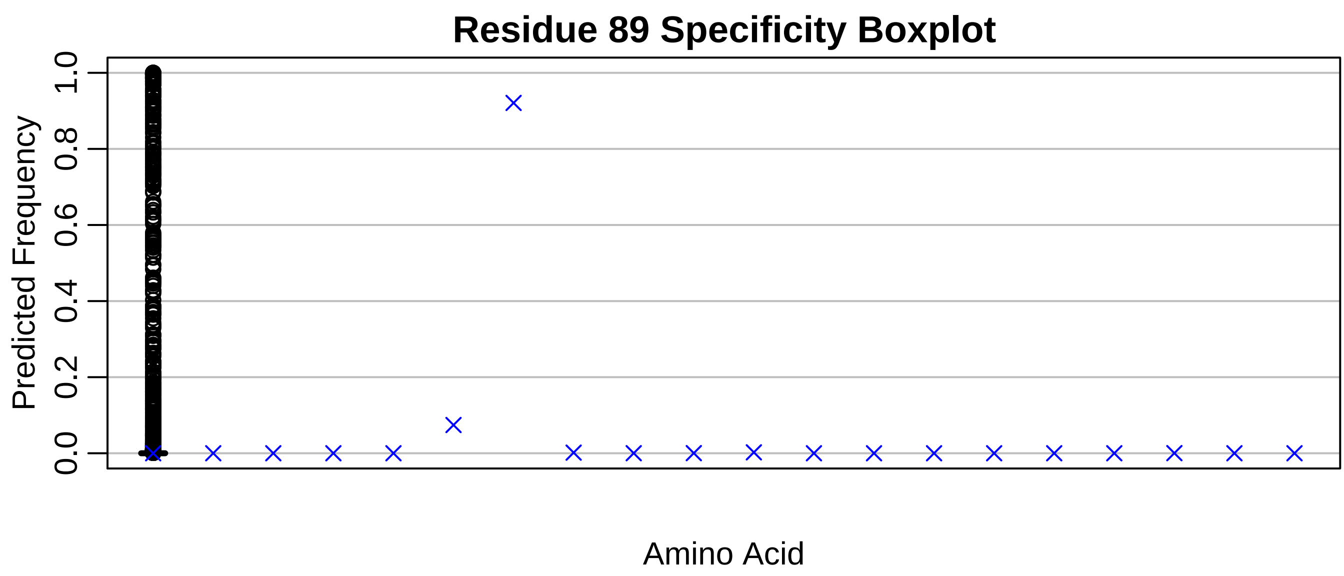

Residue 89 Specificity Boxplot
Predicted Frequency
0.0
0.2
0.4
0.6
0.8
1.0
Amino Acid
