# Supplementary material for: Predicting the Tolerated Sequences for Proteins and Protein Interfaces Using RosettaBackrub Flexible Backbone Design
Source: PLoS One. 2011 Jul 18;6(7):e20451. doi: 10.1371/journal.pone.0020451 (PMC3138746; doi:10.1371/journal.pone.0020451)
Supplement: Dataset S1 — Protocol Capture (BZ2) [file pone.0020451.s008.bz2 › DatasetS1/output_files/1N7T_V83K/specificity_boxplot.pdf]

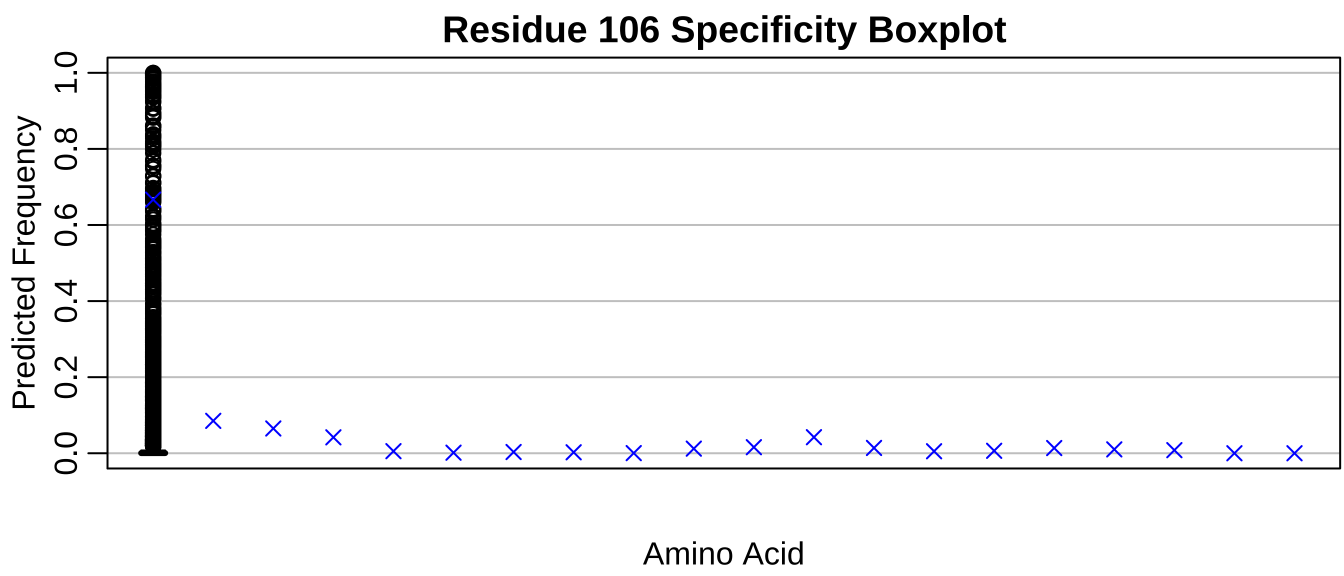

Residue 106 Specificity Boxplot
Predicted Frequency
0.0
0.2
0.4
0.6
0.8
1.0
Amino Acid

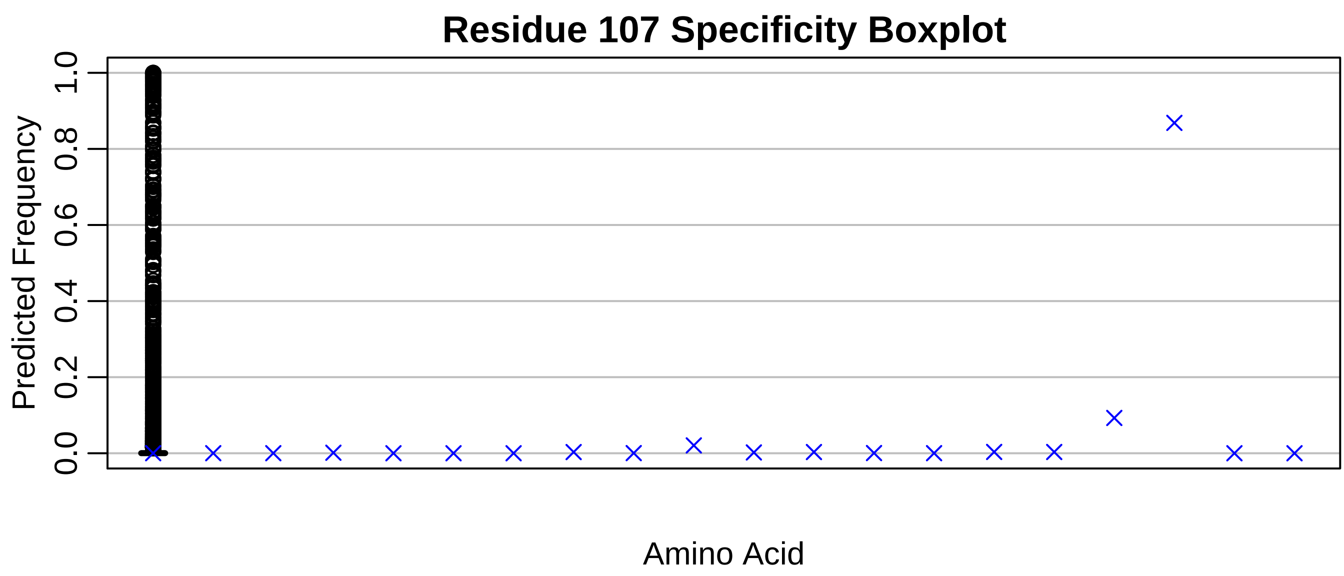

Residue 107 Specificity Boxplot
Predicted Frequency
0.0
0.2
0.4
0.6
0.8
1.0
Amino Acid

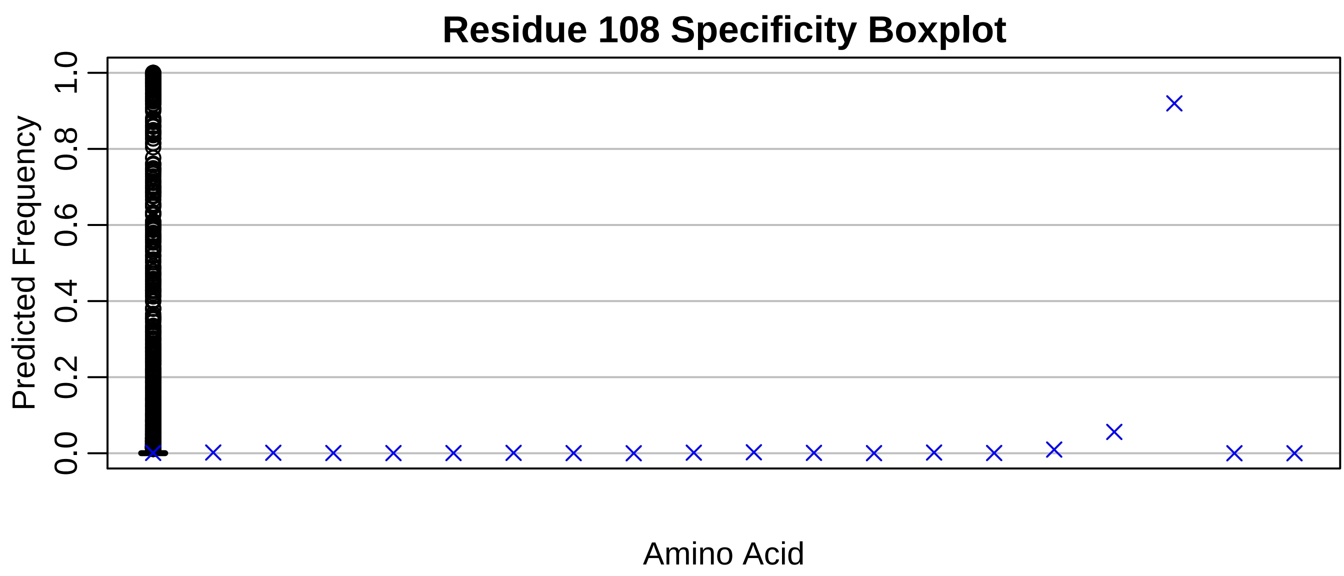
Residue 108 Specificity Boxplot
Predicted Frequency
0.0
0.2
0.4
0.6
0.8
1.0
Amino Acid

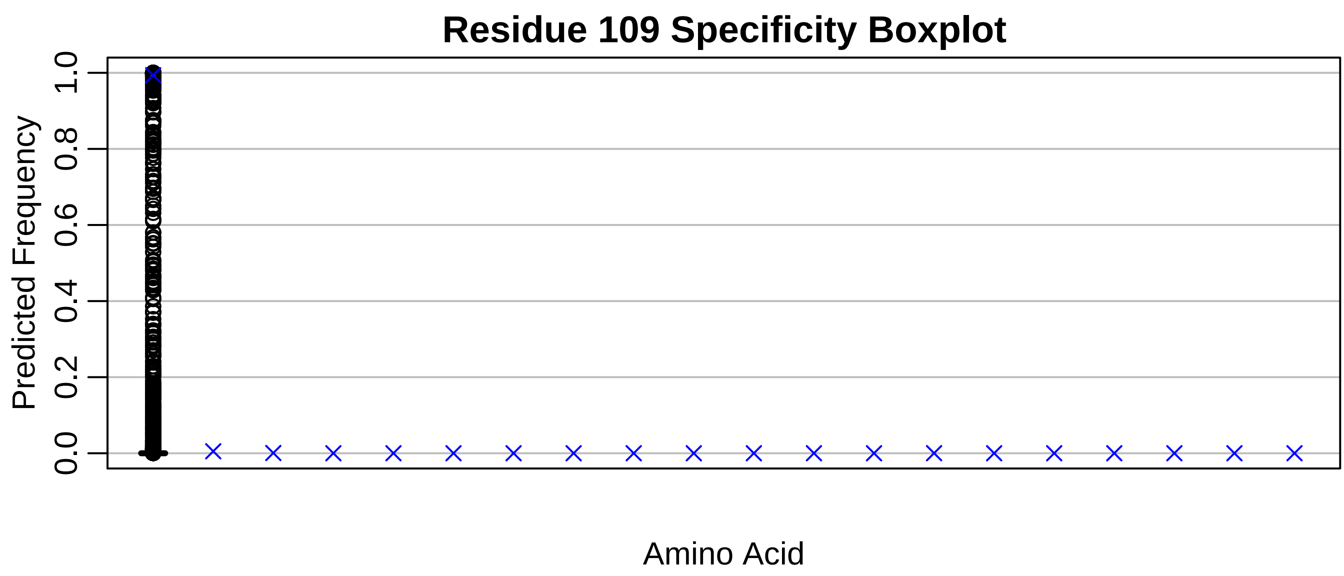

Residue 109 Specificity Boxplot
Predicted Frequency
1.0
0.8
0.6
0.4
0.2
0.0
Amino Acid

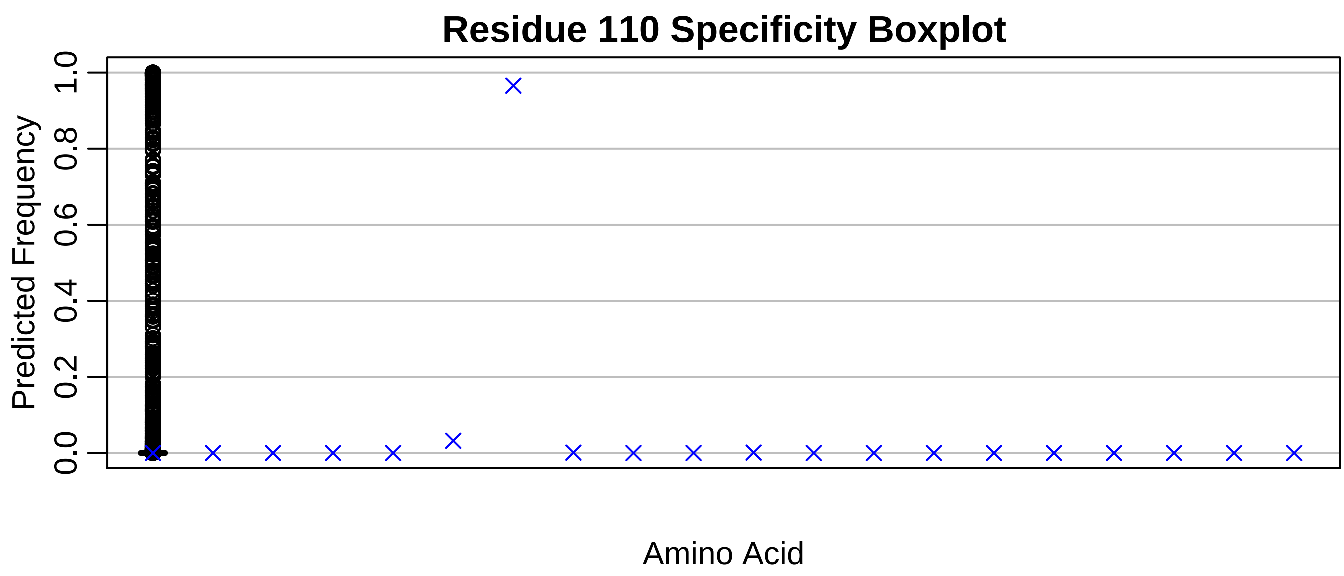
Residue 110 Specificity Boxplot
Predicted Frequency
0.0
0.2
0.4
0.6
0.8
1.0
Amino Acid
