# Supplementary material for: Predicting the Tolerated Sequences for Proteins and Protein Interfaces Using RosettaBackrub Flexible Backbone Design
Source: PLoS One. 2011 Jul 18;6(7):e20451. doi: 10.1371/journal.pone.0020451 (PMC3138746; doi:10.1371/journal.pone.0020451)
Supplement: Dataset S1 — Protocol Capture (BZ2) [file pone.0020451.s008.bz2 › DatasetS1/output_files/1A22_2/specificity_boxplot.pdf]

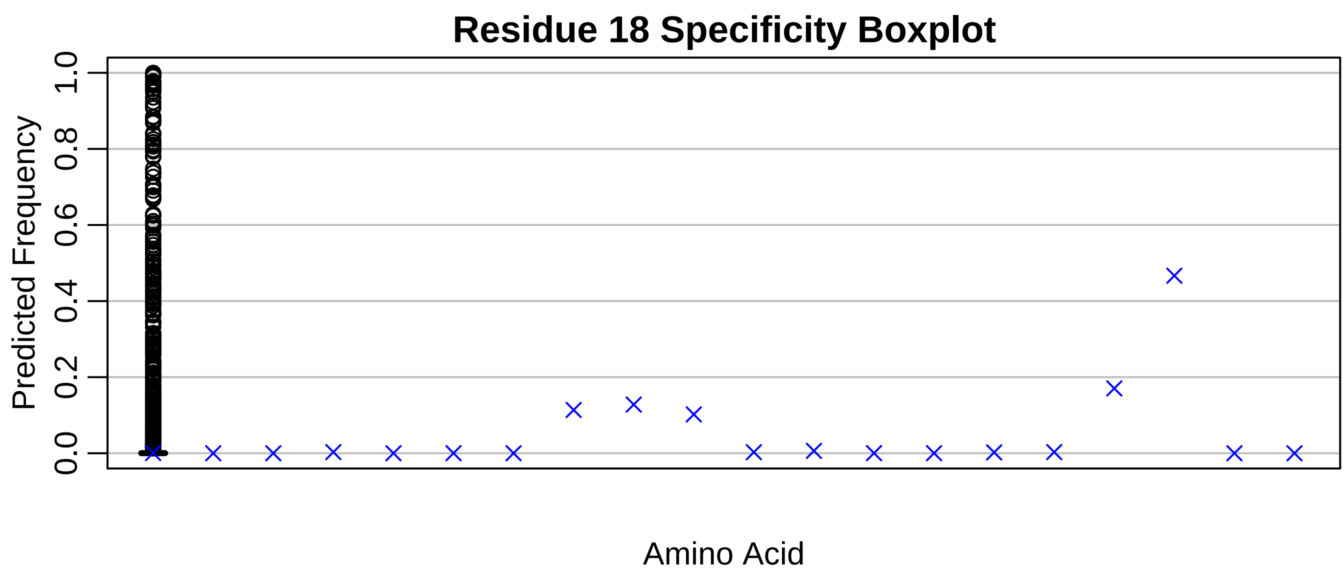

Residue 18 Specificity Boxplot
Predicted Frequency
0.0
0.2
0.4
0.6
0.8
1.0
Amino Acid

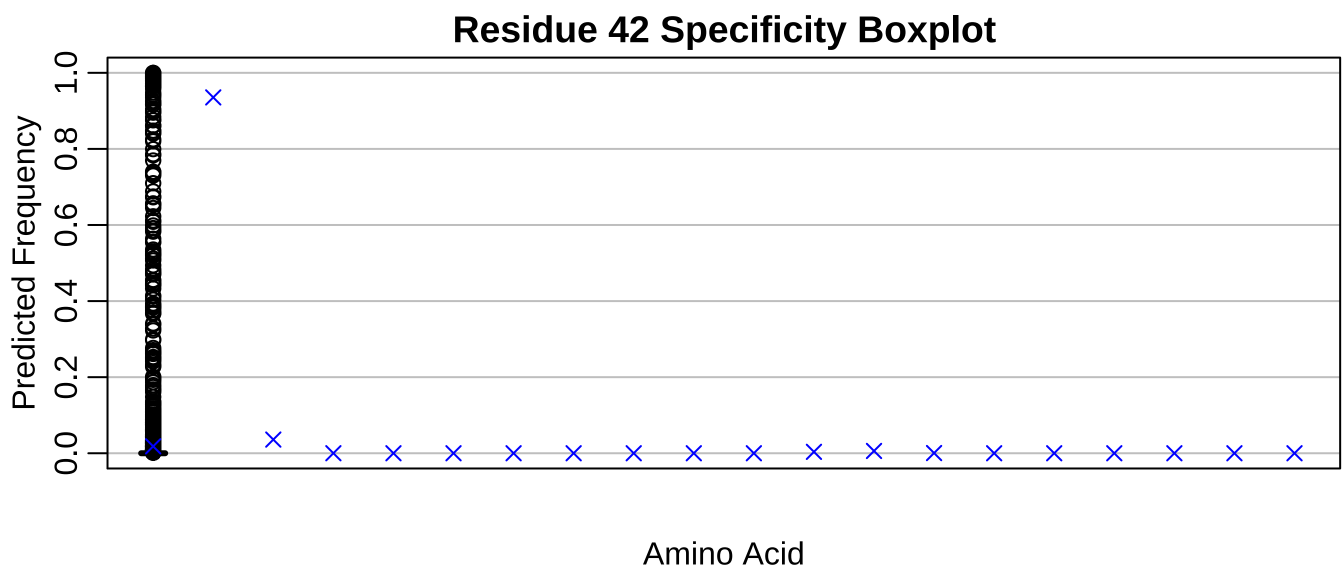

Residue 42 Specificity Boxplot
Predicted Frequency
0.0
0.2
0.4
0.6
0.8
1.0
Amino Acid

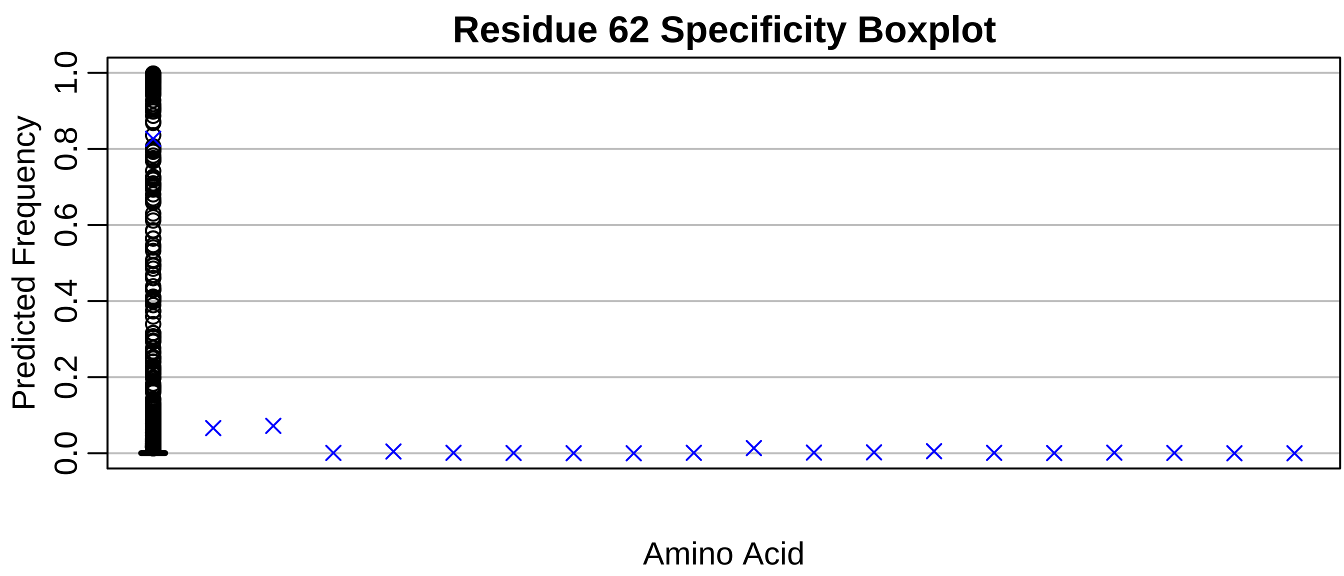

Residue 62 Specificity Boxplot
Predicted Frequency
0.0
0.2
0.4
0.6
0.8
1.0
Amino Acid

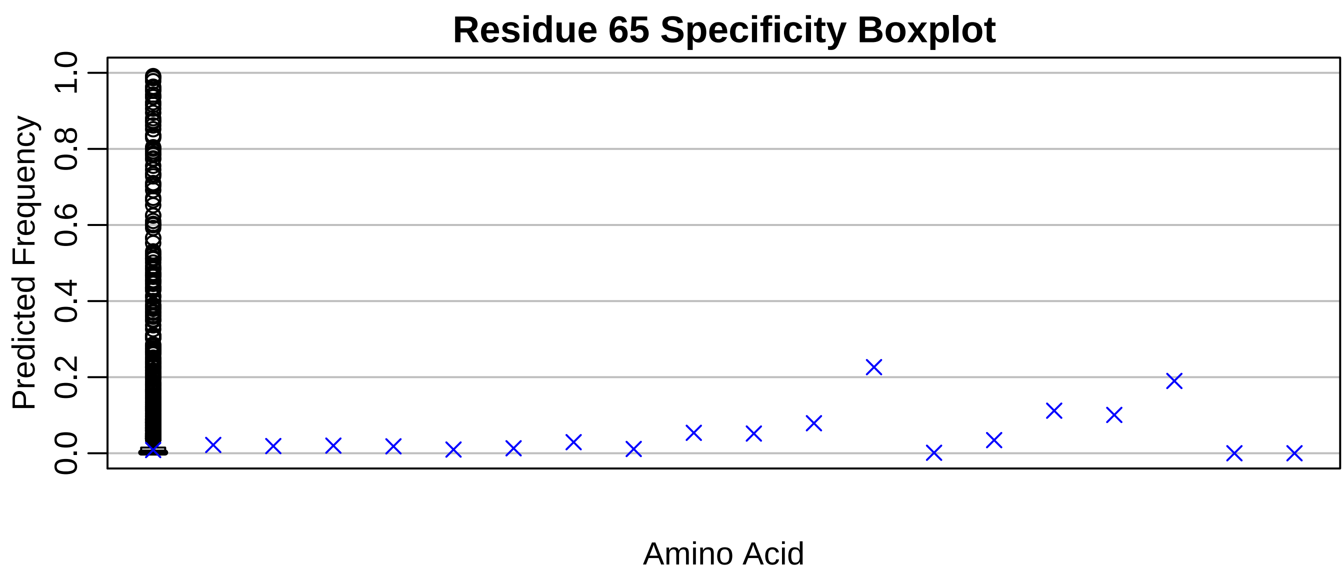

Residue 65 Specificity Boxplot
Predicted Frequency
0.0
0.2
0.4
0.6
0.8
1.0
Amino Acid

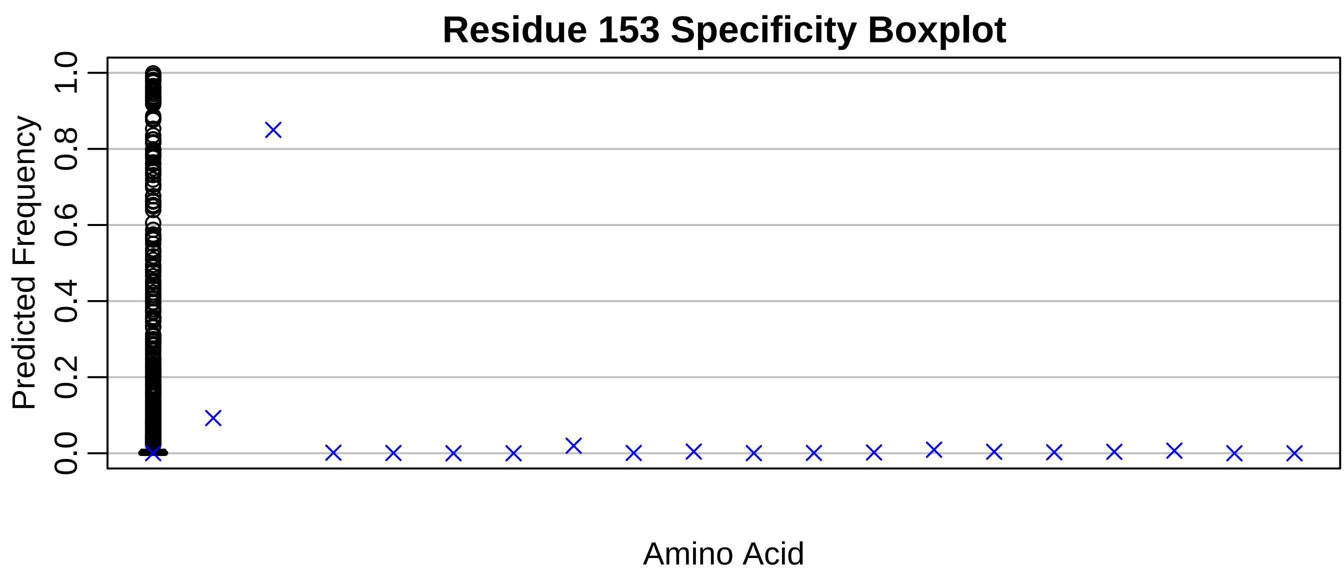
Residue 153 Specificity Boxplot
Predicted Frequency
1.0
0.8
0.6
0.4
0.2
0.0
Amino Acid

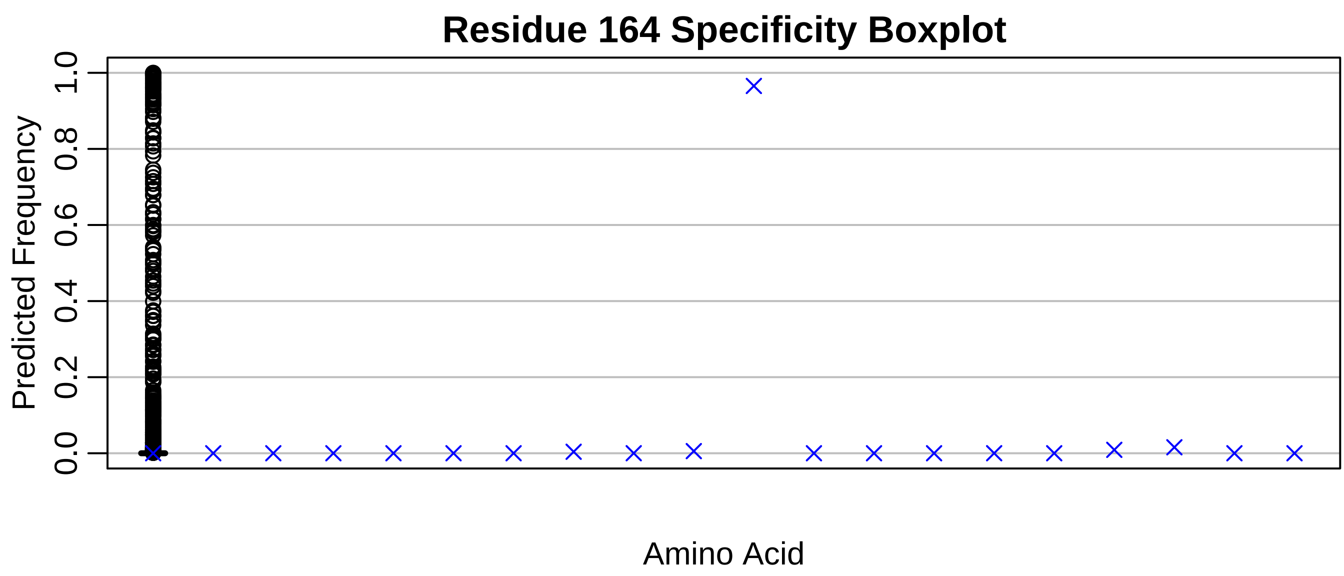

Residue 164 Specificity Boxplot
Predicted Frequency
0.0
0.2
0.4
0.6
0.8
1.0
Amino Acid
